# Parent-of-origin effects propagate through networks to shape metabolic traits

**DOI:** 10.7554/eLife.72989

**Published:** 2022-03-31

**Authors:** Juan F Macias-Velasco, Celine L St Pierre, Jessica P Wayhart, Li Yin, Larry Spears, Mario A Miranda, Caryn Carson, Katsuhiko Funai, James M Cheverud, Clay F Semenkovich, Heather A Lawson

**Affiliations:** 1 Department of Genetics, Washington University School of Medicine Saint Louis United States; 2 Department of Medicine, Washington University School of Medicine Saint Louis United States; 3 Diabetes and Metabolism Research Center, University of Utah Salt Lake City United States; 4 Department of Biology, Loyola University Chicago United States; Indiana University United States; University of Zurich Switzerland

**Keywords:** parent-of-origin effect, epistasis, adipose, RNA sequencing, metabolism, complex traits, Mouse

## Abstract

Parent-of-origin effects are unexpectedly common in complex traits, including metabolic and neurological traits. Parent-of-origin effects can be modified by the environment, but the architecture of these gene-by-environmental effects on phenotypes remains to be unraveled. Previously, quantitative trait loci (QTL) showing context-specific parent-of-origin effects on metabolic traits were mapped in the F_16_ generation of an advanced intercross between LG/J and SM/J inbred mice. However, these QTL were not enriched for known imprinted genes, suggesting another mechanism is needed to explain these parent-of-origin effects phenomena. We propose that non-imprinted genes can generate complex parent-of-origin effects on metabolic traits through interactions with imprinted genes. Here, we employ data from mouse populations at different levels of intercrossing (F_0_, F_1_, F_2_, F_16_) of the LG/J and SM/J inbred mouse lines to test this hypothesis. Using multiple populations and incorporating genetic, genomic, and physiological data, we leverage orthogonal evidence to identify networks of genes through which parent-of-origin effects propagate. We identify a network comprised of three imprinted and six non-imprinted genes that show parent-of-origin effects. This epistatic network forms a nutritional responsive pathway and the genes comprising it jointly serve cellular functions associated with growth. We focus on two genes, *Nnat* and *F2r*, whose interaction associates with serum glucose levels across generations in high-fat-fed females. Single-cell RNAseq reveals that *Nnat* expression increases and *F2r* expression decreases in pre-adipocytes along an adipogenic trajectory, a result that is consistent with our observations in bulk white adipose tissue.

## Introduction

Parent-of-origin effects, where the phenotypic effect of an allele depends on whether the allele is inherited maternally or paternally, are epigenetic phenomena associated with a wide range of complex traits and diseases (Lawson et al., 2013). Thus, the functional impact of a specific genetic variant can depend on its parental origin. The best characterized parent-of-origin effect is genomic imprinting, an epigenetic process in which either the maternally or paternally inherited allele is silenced, typically through DNA methylation. In humans, there are 107 verified imprinted genes and in mice there are 124, of which ~ 70% overlap (Jirtle, 2012). Despite the rarity of imprinted genes, parent-of-origin effects on complex traits and diseases are relatively common, suggesting that canonical imprinting mechanisms are not sufficient to account for these phenomena (Mozaffari et al., 2019; Zeng et al., 2019). With so few imprinted genes, what mechanisms underlie these parent-of-origin effects? We hypothesize that a small number of imprinted genes can generate a large number of parent-of-origin effects through interactions with non-imprinted genes.

In this study, we use four populations at different levels of intercrossing of the LG/J and SM/J inbred mouse lines to test the hypothesis that non-imprinted genes can contribute to parent-of-origin effects on metabolic phenotypes through epistatic interactions with imprinted genes. Multiple populations (F_0_, F_1_, F_2_, F_16_) allow us to refine our search space and provide orthogonal evidence supporting putative networks of interacting genes. Metabolic traits were previously mapped in a F_16_ generation of an advanced intercross between LG/J and SM/J (Cheverud et al., 2011; Lawson et al., 2010; Lawson et al., 2011a; Lawson et al., 2011b). We generated visceral white adipose tissue gene expression profiles from 20 week-old F_1_ animals in order to match the age of the F_16_ LG/J x SM/J advanced intercross population. F_1_ reciprocal cross (LxS and SxL) mice were subjected to the same high and low-fat diets and phenotyping protocols as the previously-studied F_16_ mice to keep environmental contexts consistent. We identified genes showing parent-of-origin-dependent allele-specific expression (ASE), characterized interactions among these genes and biallelic genes that are differentially expressed by reciprocal cross (DE), and correlated interacting ASE and DE gene pairs with metabolic phenotypes in the F_1_ population. Pairs that significantly associated with phenotypic variation were tested for epistasis on correlated traits in the F_16_ population.

We identified an epistatic network that forms a nutritional environment responsive pathway mediated through calcium signaling. This network contributes to metabolic variation by balancing proliferation, differentiation, and apoptosis in adipocytes. The genes comprising this network jointly serve functions associated with growth in multiple tissues, which is consistent with the evolutionary hypothesis that sexual conflict underlies some parent-of-origin effects (Mochizuki et al., 1996). We focus on two key interacting genes: *Nnat* (neuronatin), a canonically imprinted gene, and *F2r* (coagulation factor II receptor), a biallelic gene showing significant DE by cross in F_1_ high-fat-fed female animals. Co-expression of these two genes associates with variation in basal glucose levels, and this association persists across generations. Further, single-cell RNAseq reveals that *Nnat* expression increases and *F2r* expression decreases in pre-adipocytes along an adipogenic trajectory, a pattern consistent both with their expression in bulk white adipose tissue and with their respective roles in adipogenesis. Our results demonstrate that incorporating orthogonal lines of evidence including genotype, allele specific expression, total gene expression, single-cell expression, and phenotype from different populations varying in their degree of intercrossing is a powerful way to identify putative mechanisms and test hypotheses underlying parent-of-origin effects on phenotype.

## Results

### Non-imprinted genes interact with imprinted genes and effect metabolic phenotypes

**Figure 1.**
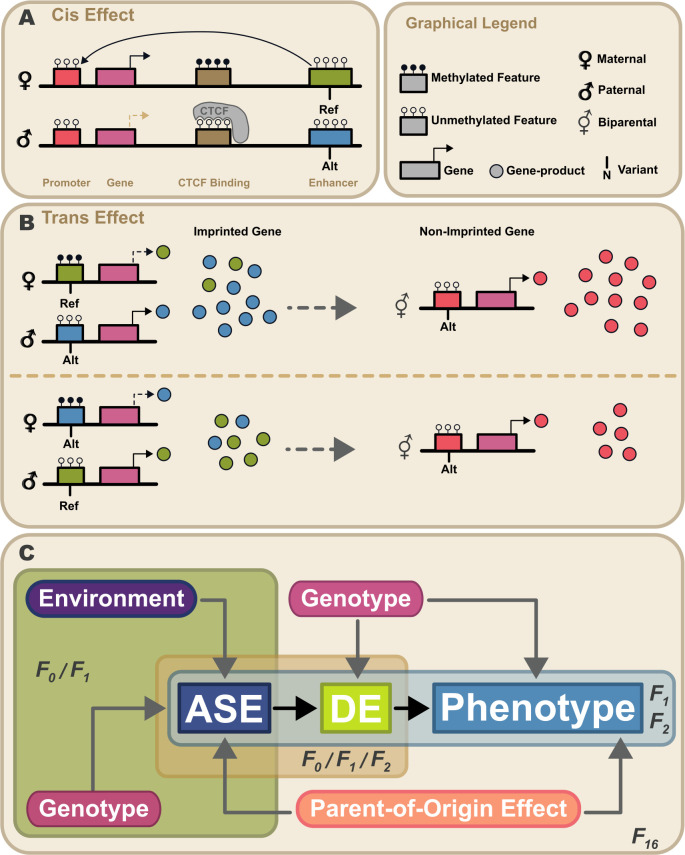
Proposed model for propagation of parent-of-origin effects through gene-gene interactions. Parent-of-origin effects should be partitioned into *cis* mechanisms and *trans* mechanisms (**A**) An example of a *cis* parent-of-origin effect is a system with three regulatory elements: promoter, insulator, and enhancer. Activation of transcription requires the enhancer to act upon the promoter. Enhancer activity is blocked by the insulator when it has been bound by CTCF. CTCF cannot be bound when methylated. In this system, the insulator is selectively methylated when inherited maternally, so methylation of the maternally inherited insulator blocks CTCF binding, allowing the enhancer to activate transcription. Because the paternally inherited insulator is not methylated, it is bound by CTCF which blocks enhancer activity, silencing transcription. This canonical genomic imprinting mechanism interacts with genetic variation in the three regulatory features. For example, if one allele produces stronger enhancer activity (Alt) than the other, individuals inheriting the Alt allele maternally would have elevated expression compared to those that inherit the same allele paternally. These *cis* genetic effects do not occur in isolation. Due to the highly interconnected nature of biological systems, there are downstream effects. We refer to these as *trans* parent-of-origin effects. (**B**) An example of a *trans* parent-of-origin effect is a system with two genes each having its own promoter. The first gene is canonically imprinted, and the activity of the gene promoter is blocked by DNA methylation. The imprinted gene’s promoter is methylated when inherited maternally. Consequently, the paternally inherited allele is almost exclusively expressed. As before, when genetic variation in a regulatory feature interacts with these epigenetic mechanisms, we see parent-of-origin effects on expression of the imprinted gene. In this example, the imprinted gene regulates expression of a non-imprinted gene. Despite the non-imprinted gene being agnostic to parental origin, its expression nonetheless depends on the parental origin of alleles at the imprinted locus. (**C**) Summary of our experimental design. Expression patterns of genes showing allele-specific expression (ASE) such as imprinted genes are shaped by parental genotypes and environment (e.g. nutrition). Downstream gene expression is a function of their genotype and the expression of upstream ASE genes. Altered parent-of-origin dependent total gene expression of ASE genes leads to differential expression of downstream genes varying only in allelic parent-of-origin (DE). Phenotype is most directly affected by expression of DE genes. Variation in DE gene expression leads to corresponding variation in phenotype. Mouse populations used to probe parts of this model are labeled F_0_ (inbred lines), F_1_ (reciprocal cross of inbred lines), F_2_ (intercross of F_1_ mice), and F_16_ (advanced intercross of inbred lines).

We test the hypothesis that non-imprinted genes can mediate complex parent-of-origin effects on phenotypes through genetic interactions with imprinted genes using a F_1_ reciprocal cross model of the LG/J and SM/J inbred mice (LxS and SxL). In this model the effects of parental origin on an allele can be tested directly and isolated from sequence dependent *cis*-regulatory differences. We validated our findings in LG/J and SM/J parentals (F_0_) as well as in F_2_ and F_16_ intercrosses of LGxSM (Figure 1). The parental F_0_ animals serve to anchor variation in allele-specific expression that is a function of allelic identity (L or S). Incorporating the F_2_ and F_16_ populations into our validations ensures that the interactions we observe are not solely a function of linkage in the F_1_ animals. We generated mRNA expression profiles in white adipose tissue from 20-week-old F_1_ reciprocal cross animals. These animals were subjected to the same high and low-fat diets and phenotyping protocols as the previously studied F_16_ animals (Cheverud et al., 2011; Lawson et al., 2011a, Lawson et al., 2010; Carson et al., 2020; Miranda et al., 2020). We identified two classes of genes: (1) imprinted genes and (2) non-imprinted genes with parent-of-origin effects on total expression.

**Figure 2.**
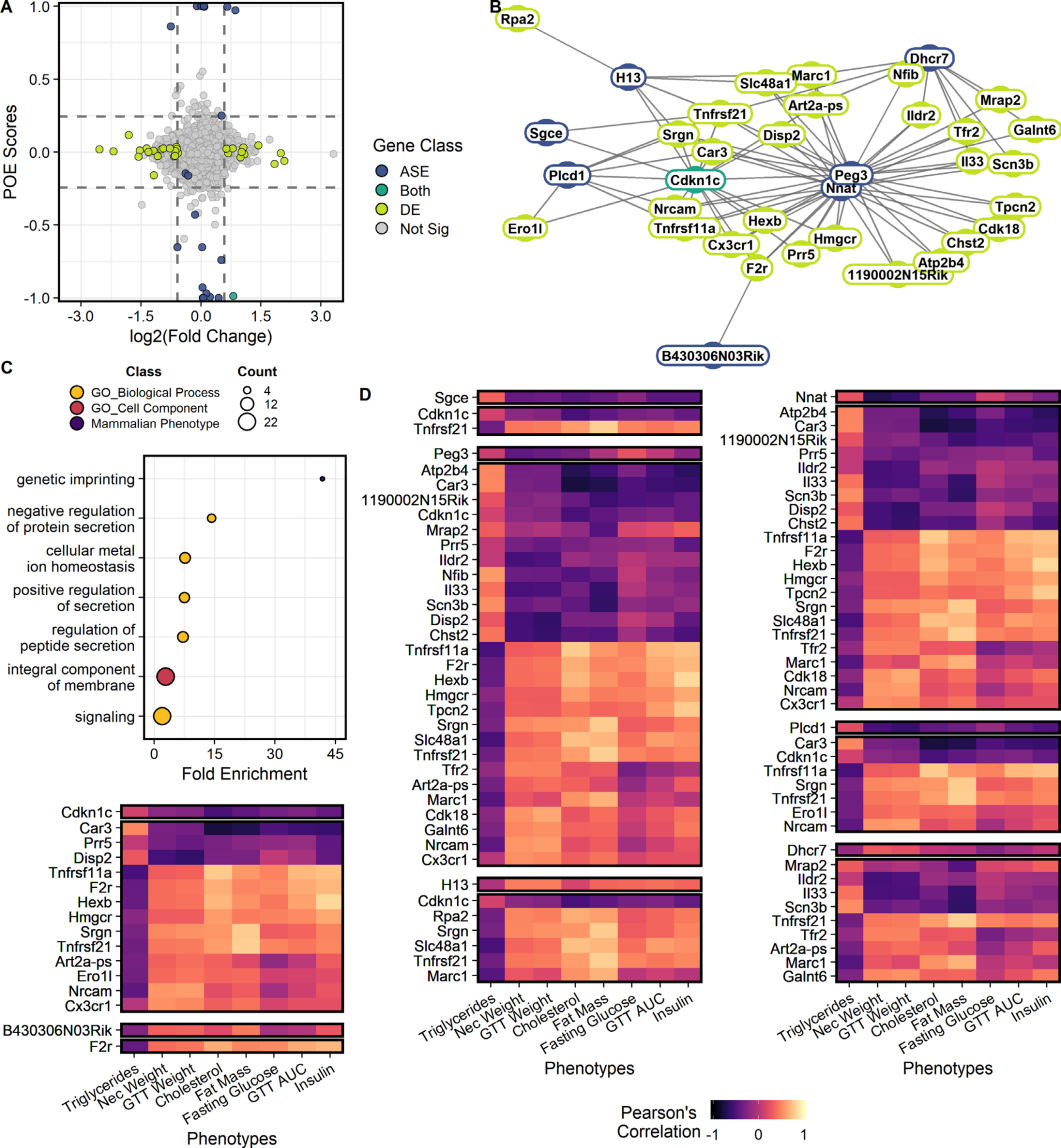
Genes showing parent-of-origin effects at the allele specific and/or total expression levels covary with each other and with metabolic traits. (**A**) Mean parent-of-origin effect score across contexts. Effect size of ASE is calculated as the mean allelic bias (L / L + S) of SxL animals minus LxS animals. Effect size of DE is measured by log_2_(Fold Change) between LxS and SxL crosses. The single context with largest magnitude fold change is plotted for each gene. Dashed lines represent minimum acceptable effect size cut-offs within a context. Genes showing significant ASE and sufficiently large parent-of-origin effect score are shown in blue. Genes showing significant DE and sufficiently large fold change in some sex or dietary context are shown in lime. Genes showing both ASE and DE are shown in teal. Genes not meeting cut-offs are shown in gray. The two genes showing significant ASE but falling short of parent-of-origin effect score requirements are a case of context dependent bipolar parent-of-origin effect scores (i.e. paternally expressed in one context and maternally expressed in its opposite). (**B**) Parent-of-origin effect network constructed from ASE and DE gene pairs. (**C**) Significantly overrepresented ontologies after multiple tests correction in the parent-of-origin effect network. Terms are color coded by ontology domain. GO biological process (yellow), GO cellular component (orange), and Mammalian phenotype (purple). Circle size denotes the number of genes with each term. (**D**) Correlation of parent-of-origin effect network genes with metabolic traits. Only genes and phenotypes with at least one significant correlation after multiple test corrections are shown. The heatmap is broken up into subnetworks with the ASE gene as the first separated row followed by associated DE genes in subsequent rows. Columns correspond to metabolic traits. Coloration of each cell denotes the Pearson’s correlation coefficient value.

To test our model, we identified genes showing parent-of-origin dependent allele specific expression (ASE). We identified 23 genes showing significant ASE (Figure 2A; Supplementary file 1). Of these 23 genes, 17 are canonically imprinted genes, two are not reported as imprinted genes but are located in known imprinted domains, and four are novel. Next, we identified genes showing differential total expression between individuals varying only in allelic parent-of-origin (DE between reciprocal crosses, SxL vs LxS). We identified 33 genes that are significantly DE in at least one sex or dietary context (Figure 2A; Supplementary file 2). A larger set of genes show signatures of parent-of-origin effects at the total gene expression level, but do not meet the statistical rigor demanded by the massive multiple tests burden incurred by a genome-wide scan accounting for sex, diet, and parent-of-origin (see Materials and methods).

**Figure 2—figure supplement 1.**
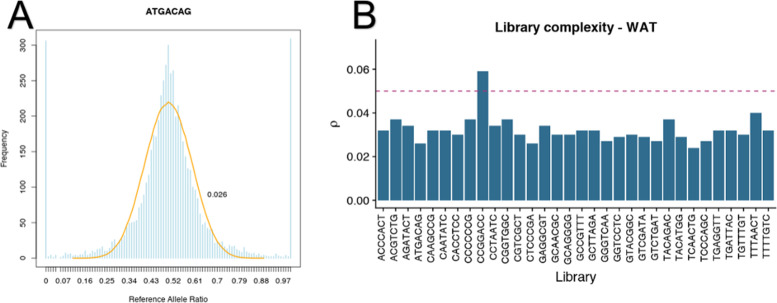
RNAseq libraries are sufficiently complex to detect allele specific expression. Complexity was measured by fitting a beta-binomial distribution to the distribution of L_bias_ values. A representativeIn this study, we use four populations at distribution (**A**) of L_bias_ values (blue) and fit beta-binomial (orange). The shape parameters (α, β) of the beta-binomial distribution are used to calculate dispersion (ρ). Dispersion value less than 0.05 indicate sufficient complexity. One library was not sufficiently complex (**B**) and was removed from analyses.

**Figure 2—figure supplement 2.**
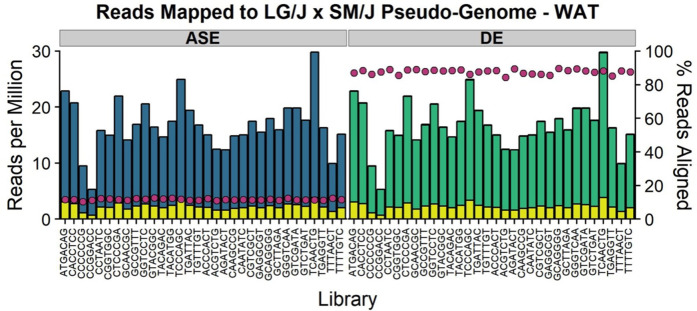
Number of reads mapped to LG/J x SM/J pseudo-genome. We isolated total RNA from white adipose (n = 32 per sex/diet/cross cohort) and constructed RNA-Seq libraries. Libraries were sequenced 1 sample per lane on an Illumina HiSeq 4000 (Illumina, San Diego, USA) using 2 × 100 bp PE reads. FASTQ files were filtered to remove low-quality reads. Remaining reads were aligned against both LG/J and SM/J pseudo-genomes simultaneously using STAR with either multimapping disallowed (allele-specific expression, ASE) or multimapping allowed (differential expression, DE). The stacked bars denotes the number of reads per million (left y-axis) and are color-coded by category (uniquely = yellow, multi-loci = green, too many = blue). For the ASE alignment, ‘too many’ reads are defined as n > 1 since multimapping was disallowed. For the DE alignment, ‘multi-loci’ reads are defined as 1 < n < 10 and ‘too many’ reads are defined as n > 10. The pink dots denote the primary alignment rates (right y-axis) for each library. Alignment rates are defined as (# reads mapped uniquely + # reads mapped multi-loci / total # input reads).

**Figure 2—figure supplement 3.**
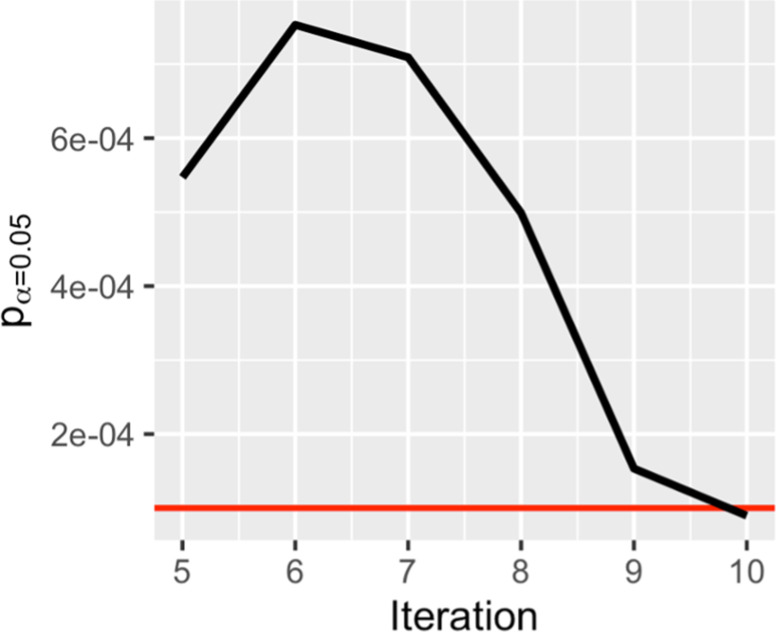
Stable null permutation plots for allele-specific expression. Context was permuted to generate an appropriate null model of p-values. Each iteration represents one genome wide permutation where every gene (7,869) was permuted separately, thus each iteration represents 7,869 separate shuffles. Null distribution stability was evaluated by calculating the critical value (in this case a significance estimate) for alpha = 0.05 at each iteration. The standard deviation of critical values was calculated after each iteration for the last 5 iterations. The model was considered stable once variation in critical values dropped below 1e-4 (red line). The variation in critical values of the null model approaches zero over ten genome wide iterations. This approximation towards zero demonstrates the stabilization of the null model. By 10 genome wide shuffles the critical value fluctuates <1e-4 units per iteration in the most complex model, meaning that at false discovery rate estimates might vary by ±1e-4.

**Figure 2—figure supplement 4.**
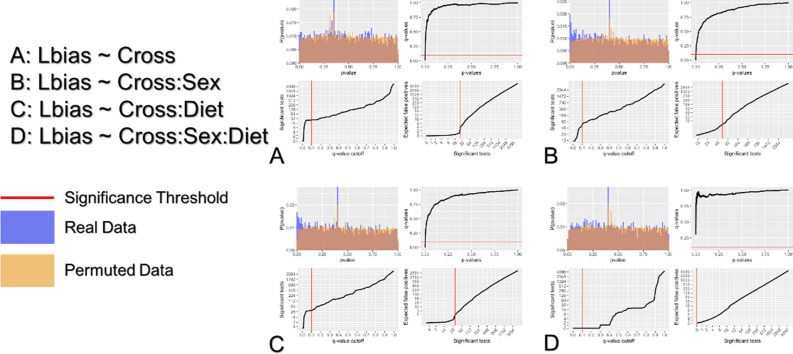
Multiple tests correction of ASE detection. Multiple tests correction was performed by estimating the false discovery rate using a permuted null model of p-values. Context was permuted to generate an appropriate null model of p-values. The distribution of permuted (orange) and real (blue) p-values is shown for each term in the model in the top left of each subplot. False discovery rate was estimated as the proportion of tests below a given significant threshold under the null relative to the real data (Null/Real). A ratio of 1 meaning that ~100% of observations at that significance threshold are likely false discoveries. The relationship between false discovery rate and p-value threshold is shown in the top right subplot for each mode. The number of significant tests at a given acceptable false discovery rate is shown as the bottom left subplot for each model. The number of false positives relative to the number of significant tests is shown as the bottom right subplot for each model.

**Figure 2—figure supplement 5.**
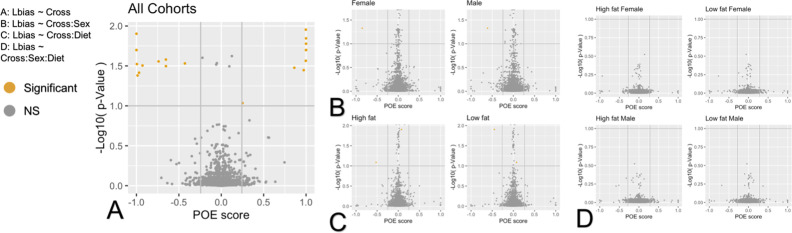
Volcano plots of parent-of-origin dependent allele-specific expression. Log_10_ transformed FDR plotted against POE score [ mean SxL Lbias – mean LxS Lbias ] for each context. Genes passing both POE score and significance thresholds are shown in orange. Genes failing to meet these criteria are shown in gray.

**Figure 2—figure supplement 6.**
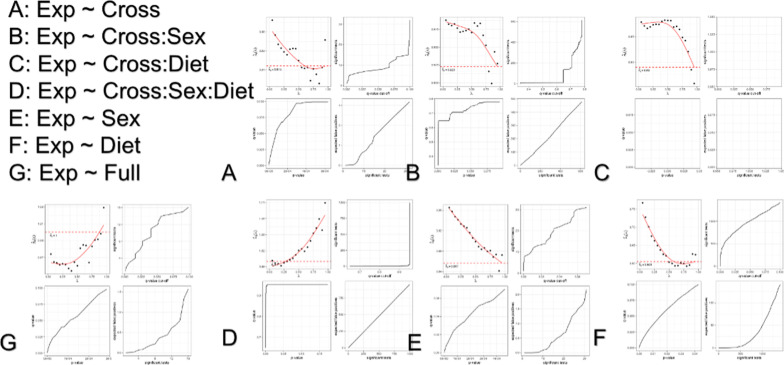
Multiple tests correction of DE detection. Multiple tests correction was performed by estimating the false discovery rate using the ‘qvalue’ R package without the generation of a permuted null model. The underlying null model is selected by the model by controlling $\hat{\pi_{\text{0}}}$. False discovery rate (q-value) is estimated as the proportion of tests below a given significant threshold derived from the estimated null model relative to the real model (Null/Real). A ratio of 1 meaning that ~100% of observations at that significance threshold are likely false discoveries. The selection of $\hat{\pi_{\text{0}}}$ is shown in the top left of each subplot for each model. The relationship between false discovery rate and p-value threshold is shown in the bottom left subplot for each mode. The number of significant tests at a given acceptable false discovery rate is shown as the top right subplot for each model. The number of false positives relative to the number of significant tests is shown as the bottom right subplot for each model.

**Figure 2—figure supplement 7.**
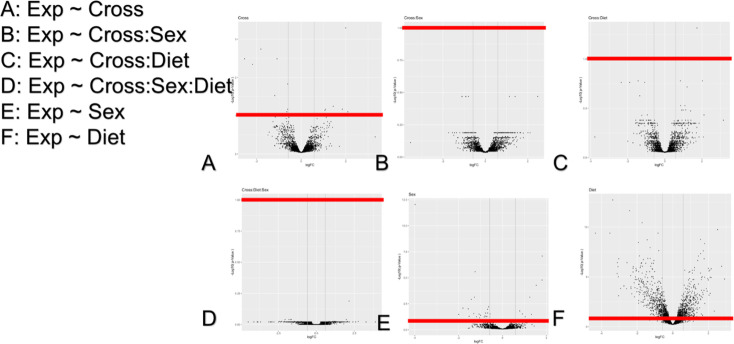
Volcano plots of differentially expressed genes. Log_10_ transformed FDR plotted against log_2_ transformed fold change for each context. The FDR threshold we used was 0.1 (red line).

**Figure 2—figure supplement 8.**
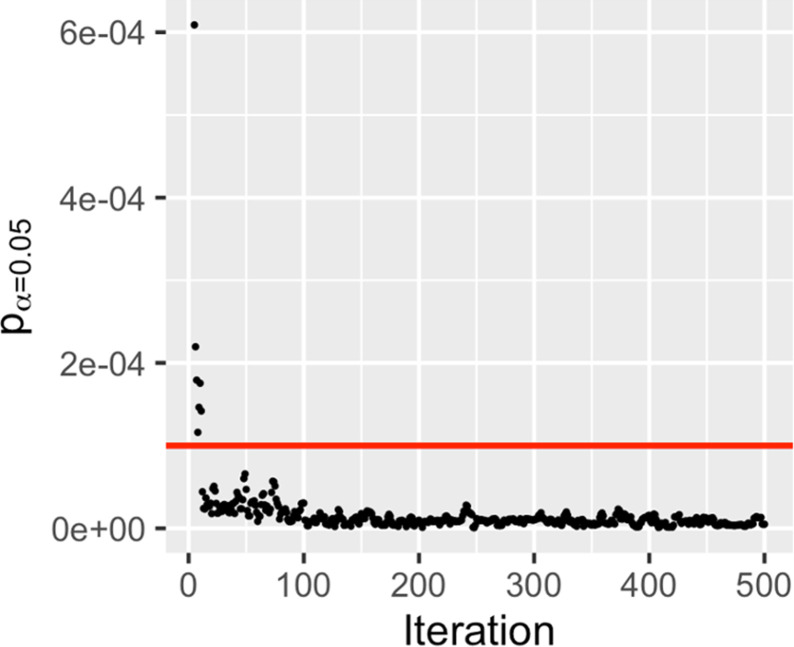
Stable null permutation plots for network pairs. Context and expression were permuted to generate an appropriate null distribution of p-values. Significance for each pair was estimate by likelihood ratio test using a chi-squared approximation. Null distribution stability was evaluated by calculating the critical value (in this case a significance estimate) for alpha = 0.05 at each iteration. The standard deviation of critical values was calculated after each iteration for the last 5 iterations. The model was considered stable once variation in critical values dropped below 1e-4 (red line). The variation in critical values under the null model approaches zero over 100 iterations. By 500 shuffles the critical value fluctuates well below 1e-4 units per iteration in the most complex model, meaning that at worst FDR estimates would vary by well below ±1e-4.

**Figure 2—figure supplement 9.**
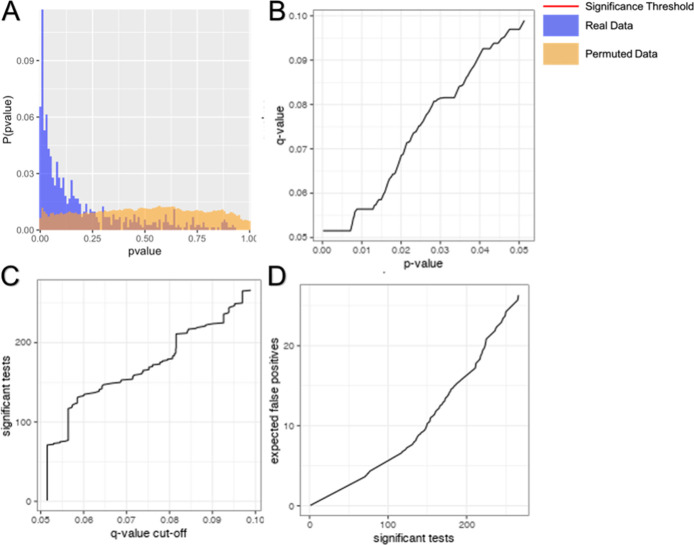
Multiple tests correction of pairwise network construction. Multiple tests correction was performed by estimating the false discovery rate using a permuted null model of p-values. The distribution of permuted (orange) and real (blue) p-values is shown (**A**). False discovery rate was estimated as the proportion of tests below a given significant threshold under the null relative to the real data (Null/Real). A ratio of 1 meaning that ~100% of observations at that significance threshold are likely false discoveries. The relationship between false discovery rate and p-value threshold is shown (**B**). The number of significant tests at a given acceptable false discovery rate is shown (**C**). The number of false positives relative to the number of significant tests is shown (**D**).

**Figure 2—figure supplement 10.**
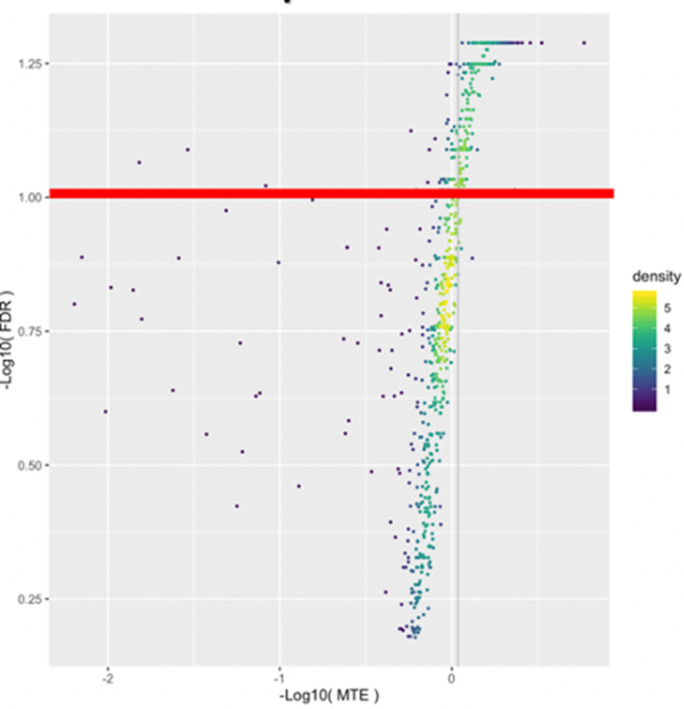
Volcano plots of network construction. Log_10_ transformed FDR is plotted against log_10_ transformed mean test error (MTE). The FDR threshold value of 0.1 is shown as the red line. The MTE threshold was set to the upper 1th percentile of the permuted model MTE and is show as the gray vertical line. Each gene is represented by a single point. Coloration of points is determined by the joint FDR/MTE density.

**Figure 2—figure supplement 11.**
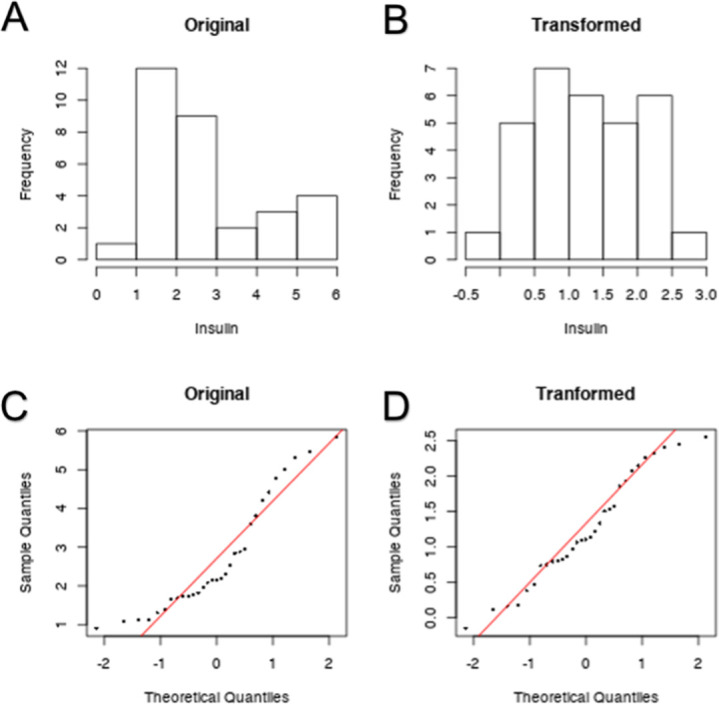
Example transformation of F_1_ phenotypes. In order to meet the assumptions of normality required by Pearson’s correlation test, non-normally distributed phenotypes were log transformed. Normality was evaluated by Shapiro-Wilkes test (p < 0.05 indicates non-normality). Serum insulin is one such phenotype. The probability distribution of serum insulin is shown before (**A**) and after transformation (**B**). This distribution is also compared to a normal distribution via quantile-quantile plot before (**C**) and after (**D**) transformation.

**Figure 2—figure supplement 12.**
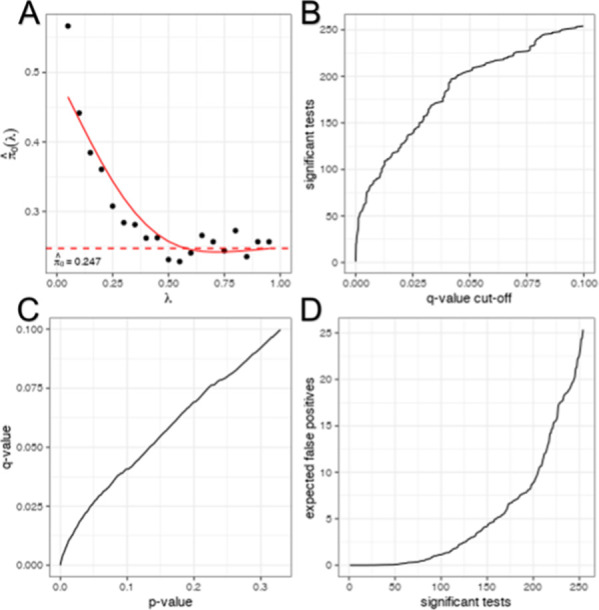
Multiple tests correction of phenotype correlations. Multiple tests correction was performed by estimating the false discovery rate using the ‘qvalue’ R package without generating a permuted null model. The underlying null model is selected by controlling $\hat{\pi_{\text{0}}}$ . False discovery rate (q-value) is estimated as the proportion of tests below a given significant threshold derived from the estimated null model relative to the real model (Null/Real). A ratio of 1 meaning that ~100% of observations at that significance threshold are likely false discoveries. Selecting $\hat{\pi_{\text{0}}}$ (**A**) allows us to determine the relationship between false discovery rate and p-value threshold (**C**). Using this relationship, we can determine the number of significant tests at a given acceptable false discovery rate (**B**). From this we can determine the number of false positives relative to the number of significant tests (**D**).

**Figure 2—figure supplement 13.**
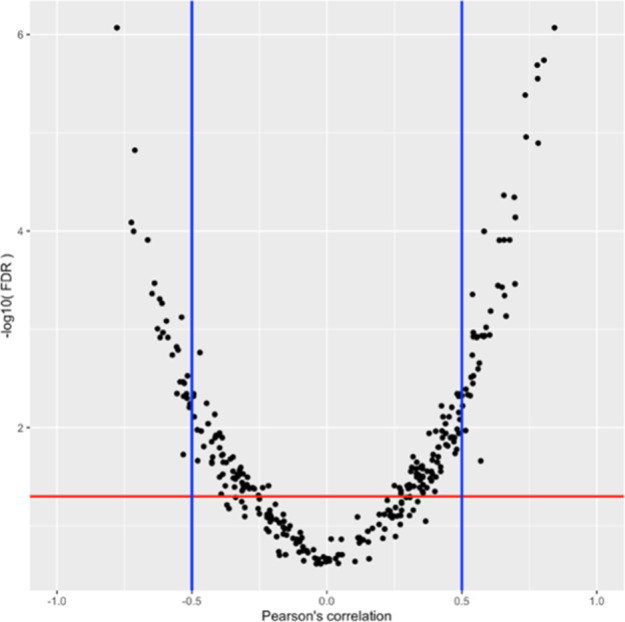
Volcano plots of phenotypes correlated with POE net gene expression. Log_10_ transformed FDR plotted against Pearson’s correlation coefficient. Each dot represents a phenotype/gene pair. The FDR threshold was set to 0.1 (red line). The correlation coefficient threshold was set to |0.5| (blue line).

To identify interactions between gene sets, we constructed a network comprised of genes that could initiate a parent-of-origin effect on phenotype (ASE) and genes that may mediate the effect onto phenotype (DE). Interacting gene pairs were predicted by modeling the expression of biallelic genes that are significantly DE by reciprocal cross as a function of the expression of genes showing significant parent-of-origin-dependent ASE, their allelic bias (L_bias_), diet, sex, and the diet-by-sex interaction. Genes showing parent-of-origin effects form a highly interconnected network comprised of 52 genes forming 217 gene pairs (Figure 2B)(Supplementary file 3). Most of these interactions are *trans*-chromosomal. We identified two genes that could serve as initiation points of propagating parent-of-origin effects through this network. These two genes, *Nnat* (neuronatin) and *Cdkn1c* (cyclin dependent kinase inhibitor 1 C), are both canonically imprinted and differentially expressed by reciprocal cross (Supplementary file 1).

Functional over representation analysis was performed and seven terms were significantly overrepresented at an FDR ≤ 0.05 (Figure 2C; Zhang et al., 2005). Enriched terms suggest this network plays a role in signaling and genetic imprinting (Supplementary file 4). In order to identify which phenotypes might be affected by genes in this network, gene expression was correlated with metabolic phenotypes collected for the F_1_ animals (Figure 2D). Seventy-four ASE/DE/phenotype sets were identified as candidates for subsequent testing (Supplementary file 5).

### Epistasis in an F_16_ advanced intercross identifies a diet-responsive network affecting adipogenesis

**Figure 3.**
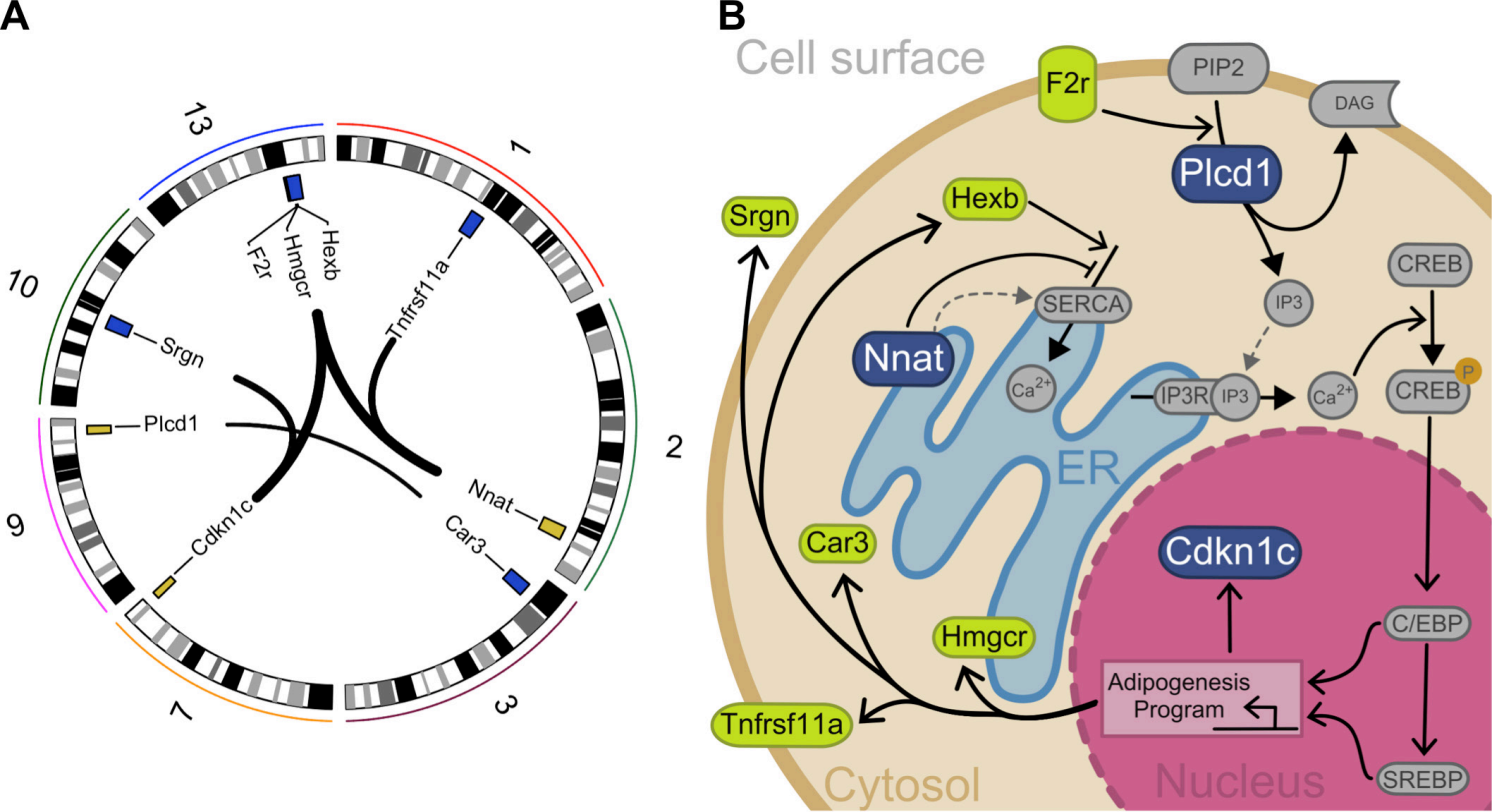
Interacting genes form a diet-responsive network affecting adipogenesis. (**A**) There are nine significant imprinting-by-imprinting epistatic ASE/DE/phenotype sets in the F_16_ advanced intercross population (n = 1002). Interactions are shown as lines connecting ASE (yellow) and DE genes (purple). Chromosome number is shown around the plot. (**B**) The epistatic parent-of-origin effect network is comprised of key steps in a putative pathway regulating differentiation and survival of adipocytes. This pathway was constructed by incorporating previously published cellular functions. The pathway members are color coded in blue for ASE genes (*Plcd1*, *Nnat*, and *Cdkn1c*) and green for DE genes (*F2r*, *Hexb*, *Hmgcr*, *Car3*, *Tnfrsf11a*, and *Srgn*). The network breaks down into potentiation, transduction, and response. *Nnat* and *Hexb* potentiate signaling by managing availability and accumulation of calcium necessary for signal transduction. Once a signal is received, *F2r* and *Plcd1* transduce it by activating second messengers to initiate a response. This response initiates an adipogenesis cellular program that affects expression of *Cdkn1c*, *Hmgcr*, *Car3*, *Tnfrsf11a*, and *Srgn*.

To validate the interactions we identified in F_1_ animals, we tested for imprinting-by-imprinting epistasis in an F_16_ population. Imprinting-by-imprinting epistasis occurs when the parent-of-origin effect at a locus is dependent on the parent-of-origin of alleles at another locus. This allowed us to determine if the effect of parent-of-origin at DE genotype on phenotype is dependent upon the parent-of-origin at ASE genotype. This orthogonal approach allows us to connect genotype at these loci to phenotype as predicted in the F_1_ candidates. Nine epistatic interactions replicated in the F_16_ population (n = 1002 animals, FDR ≤ 0.1; Figure 3A; Supplementary file 6). These interactions were comprised of three ASE genes showing parent-of-origin (*Cdknlc*, *Nnat*, *Plcd1*), six genes that are DE by cross (*Car3*, *F2r*, *Hexb*, *Hmger*, *Srgn*, *Tnfrsf11a*) and four phenotypes (basal glucose level, AUC calculated from a glucose tolerance test, serum cholesterol, necropsy weight). Together, these nine genes form a putative diet-responsive network affecting adipogenesis (Figure 3B).

**Figure 3—figure supplement 1.**
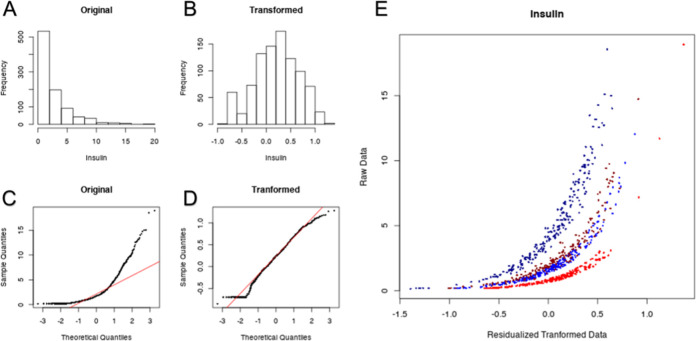
Example transformation of F_16_ phenotypes. In order to meet the assumptions of normality required by ANOVAs, non-normally distributed phenotypes were log transformed and residualized to remove the effects of sex and diet. Normality was evaluated by Shapiro-Wilkes test (p < 0.05 indicates non-normality). Serum insulin is one such phenotype. The probability distribution of serum insulin is shown before (**A**) and after transformation (**B**). This distribution is also compared to a normal distribution via quantile-quantile plot before (**C**) and after (**D**) transformation. The removal of sex and diet effects on serum insulin are shown (**E**) for high-fat males (dark blue), low-fat males (light blue), high-fat females (dark red), low-fat-fed females (light red).

**Figure 3—figure supplement 2.**
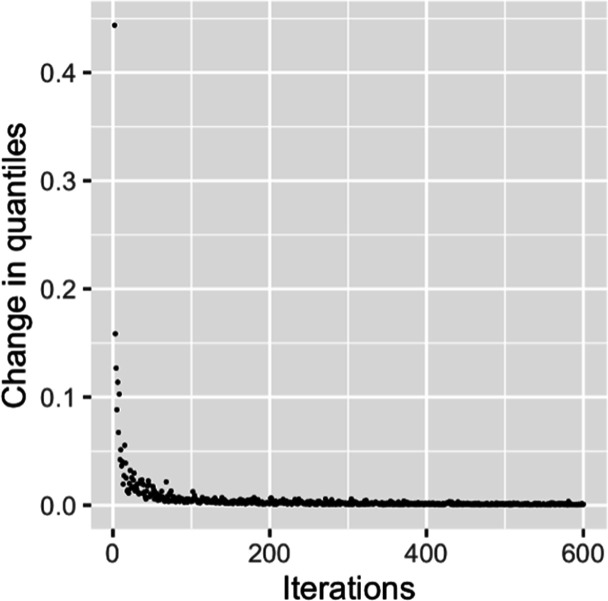
Stable null permutations plot for epistasis. Imprinting scores were permuted to generate an appropriate null model p-value. Null distribution stability was evaluated by calculating the total quantile deviation (TQD) at each iteration. The TQD of F-statistics under the null model approaches zero over 600 iterations.

**Figure 3—figure supplement 3.**
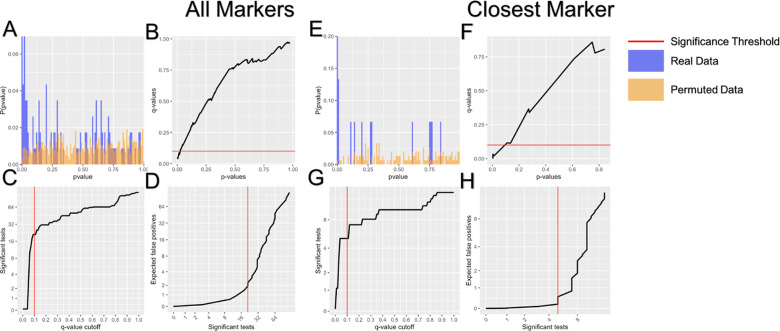
Representative multiple tests correction of imprinting:imprinting epistasis. Multiple test corrections was performed on each phenotype individually. Here were show this for the GTT area under the curve at 10 weeks phenotype using both all markers within 1.5 Mb (**A–D**) and only the nearest marker (**E–H**). Multiple tests correction was performed by estimating the false discovery rate using a permuted null model of p-values. Imprinting scores were permuted to generate an appropriate null model p-values. The distribution of permuted (orange) and real (blue) p-values is shown (A and E). False discovery rate was estimated as the proportion of tests below a given significant threshold under the null relative to the real data (Null/Real). A ratio of 1 meaning that ~100% of observations at that significance threshold are likely false discoveries. The relationship between false discovery rate and p-value threshold is shown (B and F). The number of significant tests at a given acceptable false discovery rate is shown (C and G). The number of false positives relative to the number of significant tests is shown (D and H).

The network can be broken down into signal potentiation, transduction, and response. *Nnat* (neuronatin) and *Hexb* (beta-hexosaminidase subunit beta) fall into the potentiation group. These genes play a role in managing the availability and accumulation of calcium necessary for signal transduction. *Nnat* is a paternally expressed canonically imprinted gene which encodes a proteolipid protein that localizes to the ER (Li et al., 2010). *Nnat* is diet-responsive and its overexpression in 3T3L1 pre-adipocytes promotes adipogenesis through increased free cytosolic calcium (Young et al., 2005). In pre-neural stem cells, *Nnat* binds sarco/endoplasmic reticulum Ca^2+^-ATPase (SERCA) to block Ca^2+^ uptake into the ER thereby increasing cytosolic Ca^2+^ levels (Lin et al., 2010). In addition to *Nnat*, *Hexb* regulates the uptake and accumulation of Ca^2+^ in the ER via SERCA (Pelled et al., 2003). Upon the arrival of a signal, *F2r* (coagulation factor II receptor) and *Plcd1* (1-phosphatidylinositol 4,5-bisphosphate phosphodiesterase delta-1) in the transduction group initiate the adipogenesis cellular program. *F2r* is a G-protein-bound receptor that promotes phosphoinositide hydrolysis (Soh et al., 2010). Variation in the human F2R gene is associated with obesity (Kichaev et al., 2019). G-protein-coupled receptors transmit external signals into the cell where they are then propagated by secondary messenger systems, one of which is mediated by *Plcd1* (Nakamura et al., 2005; McDonald and Mamrack, 1995). The downstream effect of *Plcd1*-mediated signaling is the efflux of calcium into the cytosol from the ER, thereby increasing cytosolic Ca^2+^ levels (Thatcher, 2010; Berridge, 2016). Increased cytosolic Ca^2+^ in pre-adipocytes promotes phosphorylation of cAMP-response element-binding protein (CREB), which promotes activity of CCAAT/enhancer-binding protein (C/EBP) transcription factors, activating adipogenesis, altering the expression of *Cdkn1c* (cyclin dependent kinase inhibitor 1 C), *Hmgcr* (3-hydroxy-3-methylglutaryl-CoA reductase), *Car3* (carbonic anhydrase 3), *Tnfrsf11a* (TNF receptor superfamily member 11 a), and *Srgn* (serglycin).

*Cdkn1c* is a canonically imprinted maternally expressed gene that inhibits cell proliferation (Kang et al., 2008). Increased expression of *Cdkn1c* is protective against diet-induced obesity in mice (Van de Pette et al., 2018), and in humans increased caloric intake results in decreased CDKN1C expression (Franck et al., 2011). *Hmgcr* is the rate-limiting enzyme in cholesterol biosynthesis (Burg and Espenshade, 2011; Jo and Debose-Boyd, 2010) and converts HMG-CoA into mevalonate, which is essential for adipocyte survival (Yeh et al., 2018). *Srgn* is an adipocytokine thought to be part of a feedback loop with *Tnfα* (tumor necrosis factor alpha)*,* mediating paracrine cross-talk between macrophages and adipocytes (Lemire et al., 2007; Imoto-Tsubakimoto et al., 2013; Schick et al., 2001; Zernichow et al., 2006). *Srgn* is known to play a role in osteoblast-mediated bone mineralization (Bigdeli et al., 2010), which along with osteoclast-driven bone deconstruction drives bone remodeling (Aubin, 1992). Osteoblasts share a lineage with adipocytes, and the quantity of osteoblasts is inversely proportional to that of marrow adipose tissue (Rodríguez et al., 2008; Prockop, 1997; Ali et al., 2005; Akune et al., 2004; Cho et al., 2011; Rosen and Bouxsein, 2006; Turner et al., 2018). *Tnfrsf11a* is a cell surface protein that regulates differentiation of osteoclasts (Nakagawa et al., 1998). Osteoprotegerin (OPG) is a decoy receptor for TNFRSF11A thereby inhibiting osteoclastogenesis and bone resorption (Matsuo et al., 2020). OPG is expressed during differentiation of 3T3L1 adipocytes (An et al., 2007). Expression of OPG is induced by *Tnfα* in 3T3L1 adipocytes and is associated with obesity in humans (Holecki et al., 2007; Erol et al., 2016; Zaky et al., 2022).

The exact function of OPG/*Tnfrsf11a* outside of osteoclastogenesis is unknown, but the function of osteoclasts is to break down bone tissue during bone resorption. Bone resorption regulates the level of blood calcium. The bioavailability of calcium in the blood potentially alters ER calcium stores, creating cross-talk between bone cells and white adipose tissue calcium signaling. Osteoclasts break down bone by acidifying mineralized bone, orchestrated by osteoblasts that have become embedded in the matrix they produce (osteocytes). Oxidative stress on osteocytes from the bone acidification process is prevented by *Car3. Car3* is an enzyme that catalyzes the conversion of carbonic acid to CO_2_ and water. Its expression in white adipose is negatively correlated with, and responsive to, long-term obesity in mice and humans (Stanton et al., 1991; Font-Clos et al., 2017). *Car3* does not protect against diet-induced obesity and is not necessary for fatty acid synthesis (Renner et al., 2017). As such its exact function in adipocytes is unknown.

### Nnat and F2r covary in white adipose tissue and their interaction associates with variation in basal glucose levels across generations

**Figure 4.**
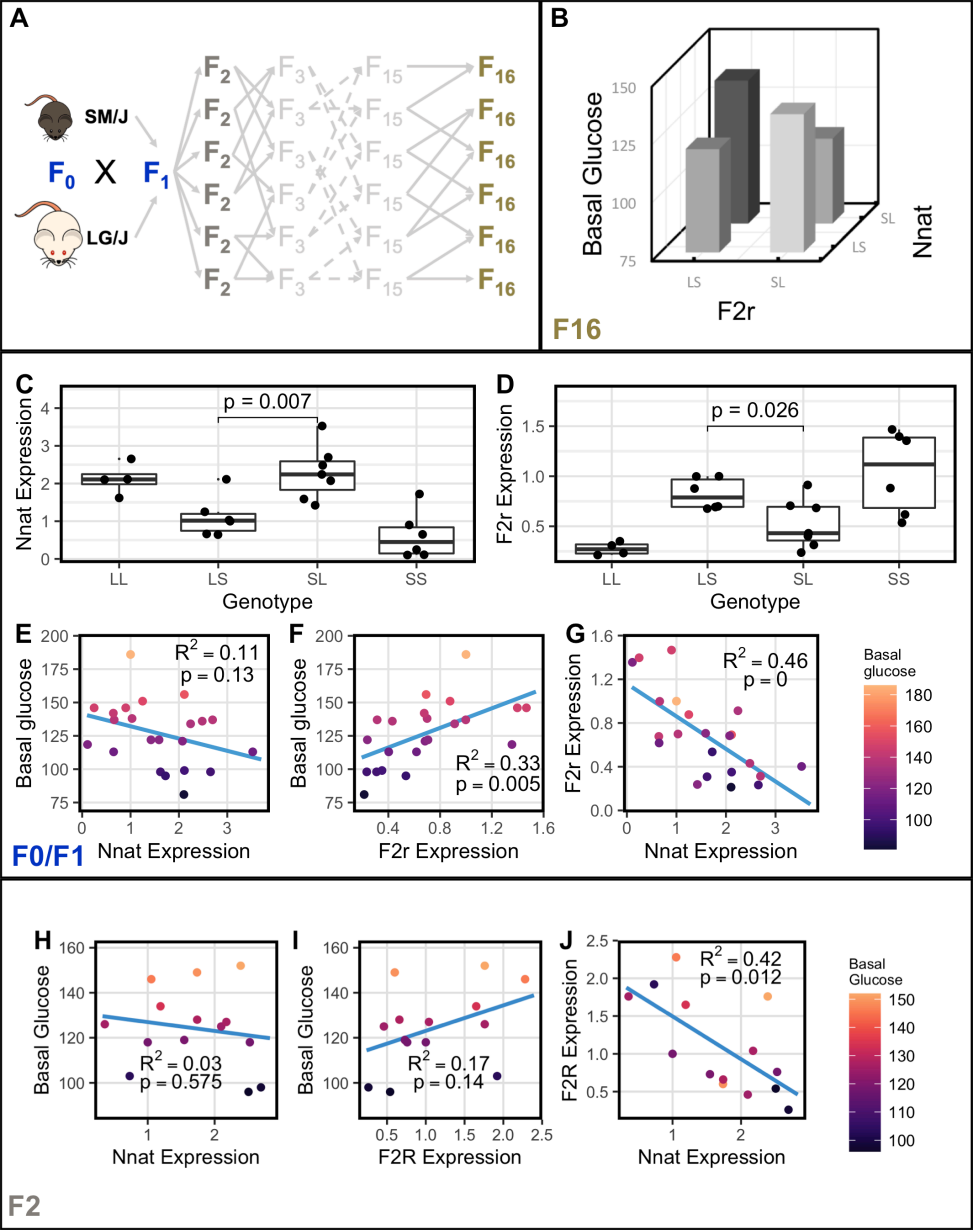
*Nnat* and *F2r* covary across generations. (**A**) Breeding scheme for the F_16_ Advanced Intercross between the LG/J and SM/J inbred strains. (**B**) Significant imprinting-by-imprinting epistasis associated with variation in basal glucose (n = 1002). The parent-of-origin effects of *F2r* on basal glucose depend on the parent-of-origin effects at *Nnat*. (**C**) Expression of *Nnat* across genotypes in a combined F_0_/F_1_ population of high fat-fed females (n = 25). (**D**) Expression of *F2r* across genotypes in a combined F_0_/F_1_ population of high-fat-fed females (n = 25). (**E**) Significant correlation between *Nnat* and *F2r* expression in the F_0_/F_1_ mice (F_1_ n = 13; F_0_ n = 12). (**F**) and (**G**) Correlations between basal glucose and *Nnat* and *F2r* in the F_0_/F_1_ mice (F_1_ n = 13; F_0_ n = 12). (**H**) Significant correlation between *Nnat* and *F2r* expression in the high fat-fed female F_2_ mice (n = 14). (**I**) and (**J**) Correlations between basal glucose and *Nnat* and *F2r* are not individually significant in the F_2_ mice. Alleles are ordered maternal | paternal within the genotype classes.

To better understand how these interactions affect phenotype, we focused on the negative correlation of the imprinted gene, *Nnat,* and the biallelic gene, *F2r,* in the above network in high fat-fed females, the cohort with the most significant covariation in the F_1_ animals (FDR = 0.05). *Nnat* and *F2r* show significant imprinting-by-imprinting epistasis for basal glucose levels in the F_16_ population (FDR = 6.00e^–16^; Figure 4A and B). To validate gene expression patterns, we combined F_1_ biological replicates and F_0_ high fat-fed female animals (F_1_ n = 13 and F_0_ n = 12) and again observe that *F2r* and *Nnat* are each significantly differentially expressed between reciprocal heterozygotes, that is by cross (Figure 4C and D). Further, the co-expression of *Nnat* and *F2r* also persists in the F_0_/F_1_ population (Figure 4E).

**Figure 4—figure supplement 1.**
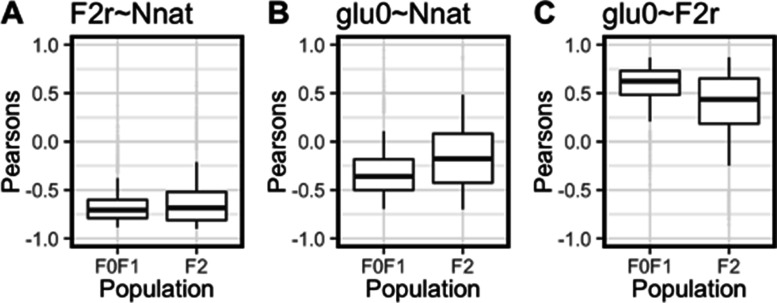
Pearson’s correlation coefficient confidence intervals. To determine whether the pattern of correlations is significantly different between F_0_/F_1_ and F_2_ populations bootstrapping was employed (N = 10, 5000 iterations). Pickets correspond to 90% confidence intervals, boxes correspond to 50% confidence intervals. Overlap of confidence intervals indicates no significant difference between populations. Correlation between F2r and Nnat expression is not significantly different (**A**). Correlation between Nnat and basal glucose is not significantly different (**B**). Correlation between F2r and basal glucose is not significantly different (**C**).

A limitation of identifying covariation patterns in F_1_ and F_0_ populations is that all loci are linked. This makes it difficult to determine which ASE genes truly co-express with DE genes. While incorporation of orthogonal F_16_ genotypes and phenotypes helps reduce false discoveries, a population with randomized genetic background for which we have expression data is needed to replicate these results. To that end, F_2_ animals were generated and *Nnat* and *F2r* gene expression levels were measured via qPCR (n = 14). We found that *F2r* and *Nnat* are significantly co-expressed in high-fat-fed female F_2_ animals (Figure 4H).

*F2r* expression significantly positively correlates with basal glucose levels in the RNA-sequenced high-fat-fed female F_1_ animals (*r* = 0.514, FDR = 0.01; Supplementary file 5). *F2r* expression is also significantly positively correlated with basal glucose in the combined F_0_/F_1_ population (Figure 4G). A negative trend between *Nnat* expression and basal glucose level is observed but not statistically significant in the combined F_0_/F_1_ animals (Figure 4F). Correlation of *F2r*’s and *Nnat*’s individual expression with basal glucose in F_2_ mice follows the same pattern as in the F_0_/F_1_’s. Bootstrapping to calculate confidence intervals shows that the correlation differences between F_0_/F_1_ and F_2_ are not significant (Figure 4I and J; Figure 4—figure supplement 1). However, the product of *Nnat* and *F2r* expression (*Nnat* x *F2r*) is significantly predictive of basal glucose (p = 0.045, R^2^ = 0.29). This indicates that expression of *Nnat* and *F2r*, as a function of their genotypes and allelic parent-of-origin, are not individually sufficient to explain variation in basal glucose levels. But together they are able to explain a significant amount of phenotypic variation. This is precisely what our epistatic model would predict.

Finally, studying the F_2_ animals allows us to determine if maternal mitochondrial ancestry contributes significantly to *Nnat* or *F2r* expression or to variation in basal glucose. We find mitochondrial genome identity does not significantly covary with *F2r* expression (p = 0.198), *Nnat* expression (p = 0.365), or basal glucose (p = 0.388).

### Single-cell RNAseq reveals that Nnat expression increases and F2r expression decreases in pre-adipocytes along an adipogenic trajectory

**Figure 5.**
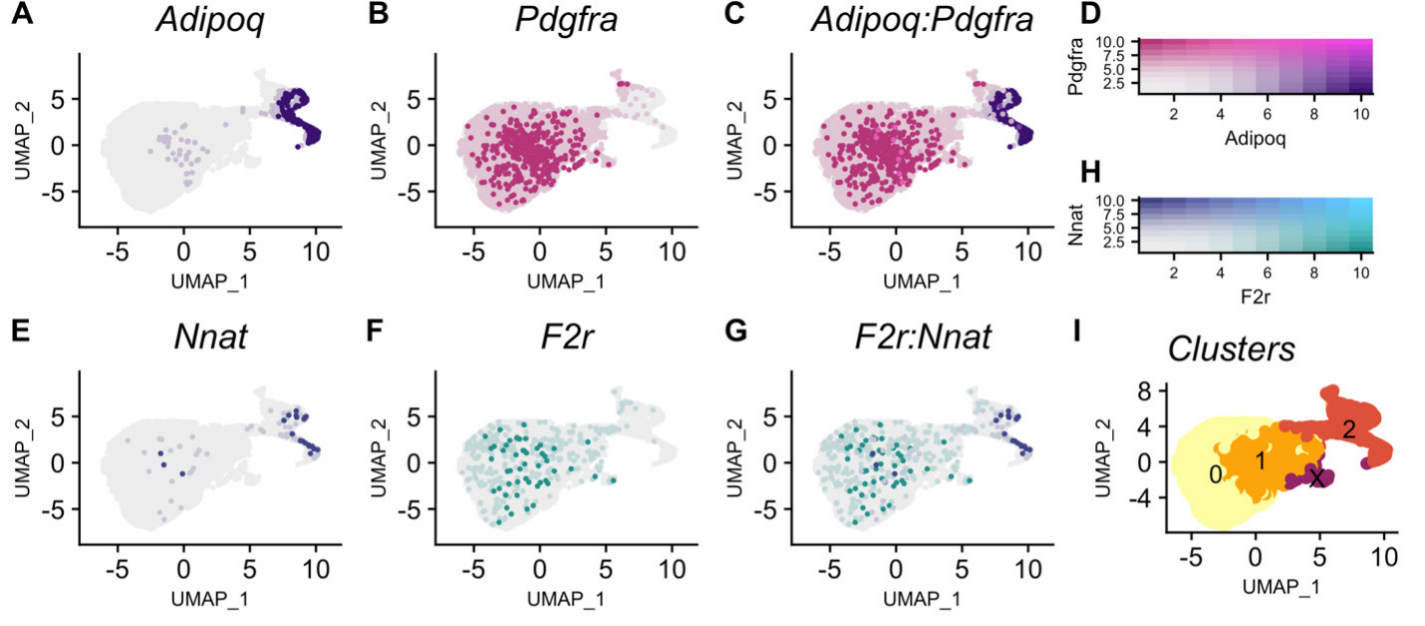
*Nnat* expression increases and *F2r expression decreases* in pre-adipocytes along an adipogenic trajectory. (**A**) *Adipoq* is a marker of adipocytes whose expression (purple) increases along the trajectory. (**B**) *Pdgfra* is a marker of mesenchymal stem cells whose expression (pink) decreases along the trajectory. (**C**) Cells in clusters expressing one or both *Adipoq* and *Pdgfra* fall along an adipogenic trajectory. (**D**) Intensity of expression of *Adipoq* and *Pdgfra* indicated by coloration. (**E**) *Nnat* expression (blue) increases along the trajectory. (**F**) *F2r* expression (teal) decreases along the trajectory. (**G**) Negative association between *Nnat* and *F2r* expression within adipocytes along the trajectory. (**H**) Intensity of expression of *Nnat* and *F2r* indicated by coloration. (**I**) The adipogenic trajectory is broken into subclusters of cells with no *Adipoq* expression (cluster 0) to high *Adipoq* expression (cluster 2).

To determine what cell types express *Nnat* and *F2r* and whether the directionality of the *Nnat* imprinted → *F2r* target correlation persists along the adipogenic trajectory, we turned to single-cell RNAseq. We used publicly available scRNAseq data collected from stromal vascular cells isolated from C57BL/6 J epididymal adipose tissue (Burl et al., 2018). Cell type identity was assigned using previously reported markers for this data set (Adipoq = differentiating mesenchymal stem cells; Pdgfra = mesenchymal stem cells; *Csf1r* = macrophage; *Cdh5* = vascular endothelial cells; *Acta2* = vascular smooth muscle cells; *Cd2* = B cells) (Supplementary file 7; Figure 5—figure supplement 1). The adipogenic trajectory refers to cells transitioning from pre-adipocytes (mesenchymal stem cells) to cells differentiating into adipocytes. Clusters along this trajectory were identified by the opposing expression patterns of *Pdgfra* and *Adipoq* (Figure 5A-D and I). We found that *Nnat* expression increases along the trajectory while F2r expression decreases (Figure 5E–F and H). Further there is a negative association between Nnat and F2r expression within adipocytes along the trajectory (Figure 5G). This pattern is consistent with the negative correlation we observe between Nnat and F2r in the bulk white adipose tissue. Because available scRNAseq data do not match the exact sex/ diet/ genetic background contexts of the LGxSM mice, there will be unaccounted for differences between the data sets. However, the observed consistent pattern indicates that the pathway structure persists across sex/ diet/ genetic backgrounds.

**Figure 5—figure supplement 1.**
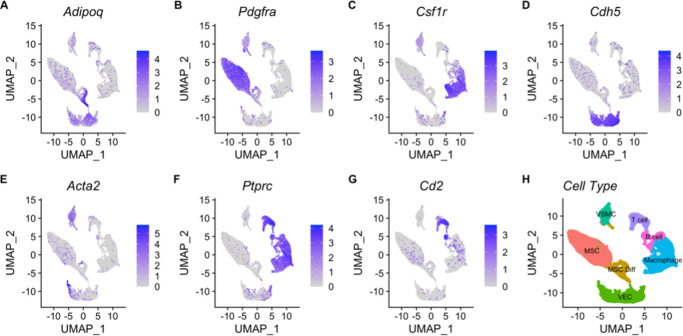
Cell types were defined using canonical markers. Expression of these markers is shown by coloration on UMAP plots for Adipoq = differentiating mesenchymal stem cells (**A**); Pdgfra = mesenchymal stem cells (**B**); *Csf1r* = macrophage(C); *Cdh5* = vascular endothelial cells(D); *Acta2* = vascular smooth muscle cells(E); Ptprc – T cells(F); *Cd2* – B cells (**G**). Cell type identities were assigned by expression of these genes (**H**).

**Figure 5—figure supplement 2.**
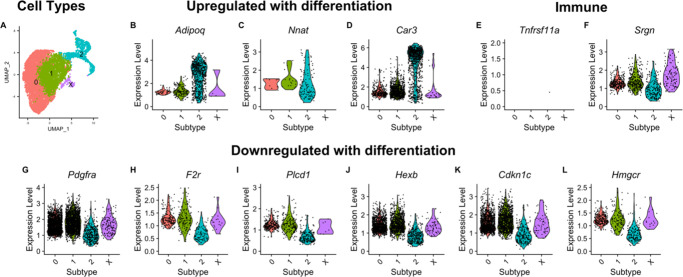
Candidate genes are differentially expressed across adipogenic trajectory. Cells along the adipogenic trajectory were clustered into 4 groups [0, 1, 2, X](**A**). Adipogenic trajectory was defined by the expression of Adipoq in differentiation MSC (**B**) and by Pdgfra in undifferentiated MSC (**G**). Expression of candidate genes identified by integrating F0/F1 RNAseq with F16 data is shown. Only cells with non-zero expression are plotted. Two candidate genes are primarily expressed by immune cells (E and F), but one of which is differentially expressed along the adipogenic trajectory (**F**). Two candidate genes are primarily expressed along the trajectory and are upregulated with differentiation (C and D). Five candidate genes are primarily expressed along the trajectory and downregulated with differentiation (**H–L**).

**Figure 5—figure supplement 3.**
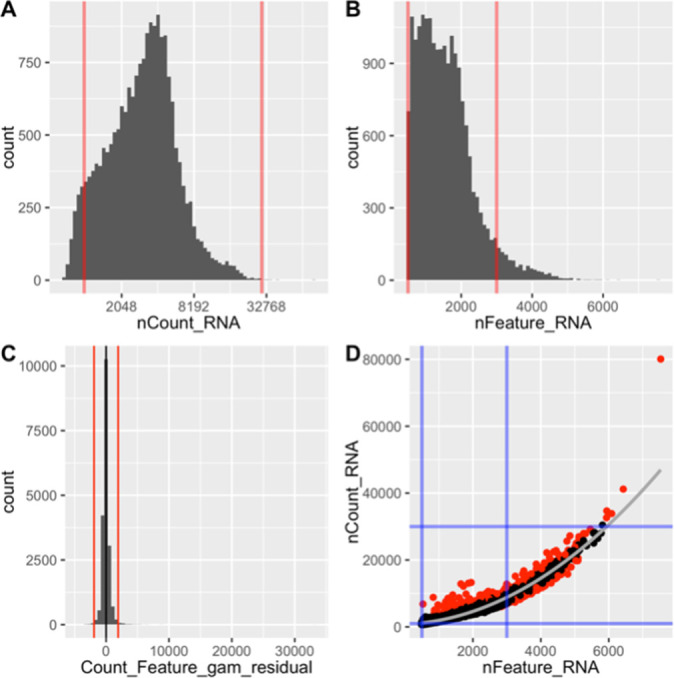
Single cell quality was controlled. The number of counts for a given gene in a given cell nCount (**A**), the number of genes expressed in a given cell nFeature (**B**), and their covariation (**C**) is predictive of cell quality. The strength of their relationship was measured as the residual for each cell in a generalized additive model predicting nCount from nFeature. Thresholds for these metrics (red/blue lines) were used to decide which cells to include. Cells with unacceptably high residuals (red dot) were thrown out.

**Figure 5—figure supplement 4.**
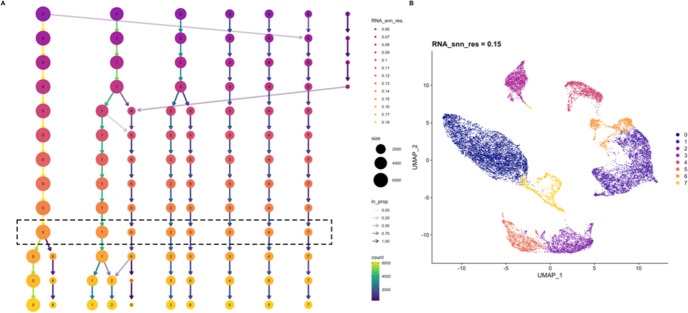
Determining the resolution for clustering. Dimensionality reduction of high dimensional single-cell RNAseq data requires the selection of a resolution parameter. This parameter is selected by seeing how stable cluster number and assignment is across a range of resolution parameter values as shown on a cluster tree (**A**). Here, we selected the highest resolution parameter that stably clustered the expected cell populations. The selected value was 0.15. Using this value, dimensionality reduction is visualized using the UMAP method (**B**). Cluster identifiers is consistent between cluster tree and UMAP projection.

**Figure 5—figure supplement 5.**
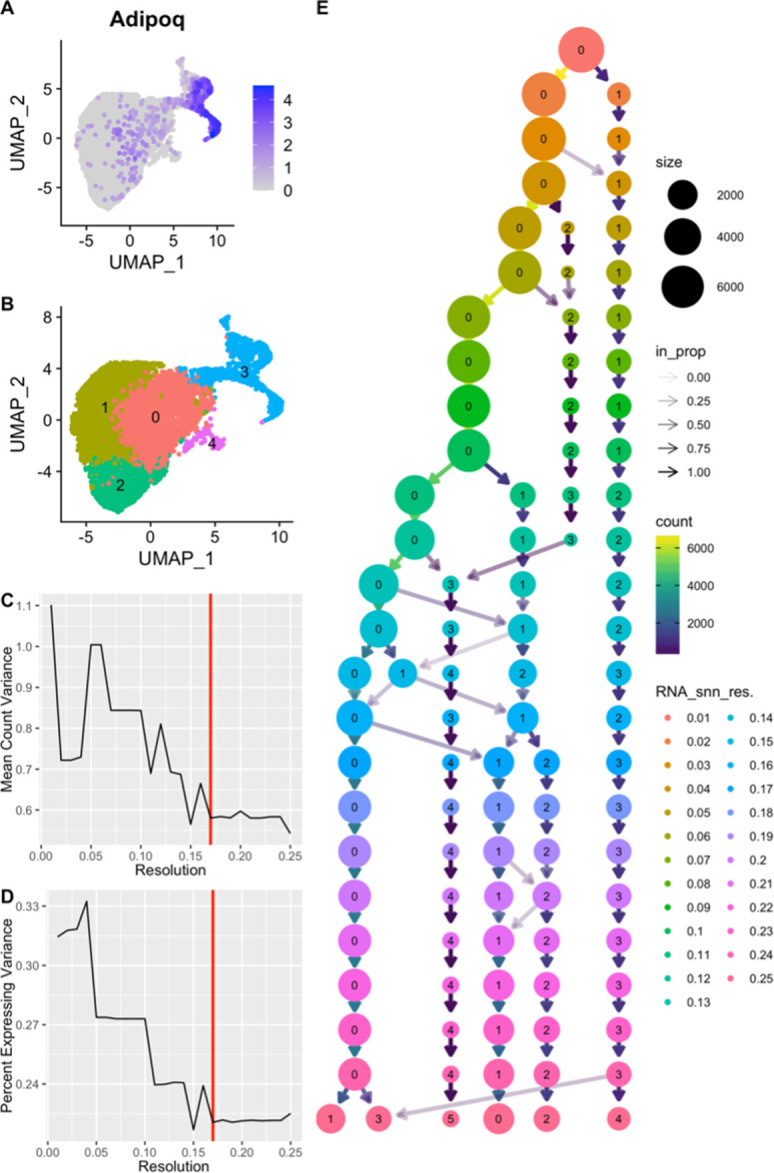
Adipocyte clustering resolution was selected to minimize Adipoq variation. We sought to test for differential expression of genes across the adipogenic trajectory. Cells from the trajectory were subset and re-clustered. We sought to cluster cells into groups that best corresponded to the different stages of differentiation (**B**). To this end we used Adipoq expression as a marker of differentiation (**A**). This dimensionality reduction of high dimensional single-cell RNAseq data requires the selection of a resolution parameter. We tested a range of resolution parameters from 0.01 to 0.25 and observed how resolution affected clustering (**E**). For each resolution parameter, the variation in mean variation in non-zero Adipoq expression within all clusters was calculated (**C**). The same was done to calculate the mean variation in percent of cells with expression >0 within all clusters. By finding the clustering conditions that in which Adipoq expression was stably minimized, we were able to select the resolution parameter that best clustered cells into the different stages of differentiation (resolution = 0.17). Dimensionality reduction is visualized using the UMAP method (**B**). Cluster identifier is consistent between cluster tree and UMAP projection.

In addition to interrogating *Nnat* and *F2r* in single cells along an adipogenic trajectory, we found that eight of the nine genes comprising the epistatic parent-of-origin effect network described above are differentially expressed along the trajectory, and they associate with cell types that are consistent with their respective roles in adipose tissue (Figure 5—figure supplement 2).

## Discussion

Epistatic interactions between imprinted and non-imprinted genes can influence complex traits when the genotypic effects of one gene depends on the parent-of-origin of alleles at another (Lawson et al., 2013; Wolf and Cheverud, 2009). Here, we examined epistatic interactions associated with parent-of-origin effects on dietary-obesity traits in white adipose using a simple yet powerful F_1_ reciprocal cross mouse model. Although these parent-of-origin dependent allele-specific expression biases are consistent with imprinting mechanisms, we cannot rule out that maternal and/or paternal effects also contribute to the phenomena we observe (Hager et al., 2008).

Interactions between imprinted and non-imprinted genes have previously been shown to contribute to variation in metabolic phenotypes. For example, the maternally expressed transcription factor KLF14 (kruppel-like factor 14) regulates biallelic gene expression related to adiposity (Parker-Katiraee et al., 2007; Small et al., 2011). Mapping studies have identified two SNPs (rs4731702, rs972283) upstream of KLF14 associated with type II diabetes and cholesterol levels (Voight et al., 2010; Teslovich et al., 2010). Both variants have maternally restricted *cis*-regulatory associations with KLF14 expression in adipose tissue (Kong et al., 2009). eQTL analysis found that rs4731702 is also enriched for *trans*-associations with KLF family transcription factor binding sites in subcutaneous white adipose tissue, suggesting that KLF14 may be a master transcriptional regulator in adipose tissue (Small et al., 2011). Whether additional pairs of imprinted and biallelic genes are similarly co-expressed and associate with phenotypic variation remains an open question that has not been thoroughly investigated in large landmark functional genomics studies including ENCODE, GTEx, and GWAS, leaving a significant gap in our knowledge. Interactions between imprinted and biallelic genes could explain some of the observed parent-of-origin effect patterns associated with regions lacking obvious candidate genes, as described in a recent survey of 97 complex traits measured in outbred mice (Mott et al., 2014).

Our model asserts that parent-of-origin effects start at ASE genes and are transduced through DE genes onto phenotype. This is illustrated in the interaction between *Nnat* and *F2r*. If a *cis*-regulatory effect interacts with epigenetic modifications (i.e. imprinting) at *Nnat*, then *Nnat* expression of genotypic classes are affected by paternal allele identity (Lawson et al., 2013). Between the LG/J and SM/J alleles at *Nnat*, the LG/J allele is more highly expressed. If our model is correct, the downstream DE gene should show a corresponding pattern (Figure 1B). In the case of *Nnat* and *F2r*, which have strong negative correlated expression, when the LG/J allele is inherited paternally at *Nnat*, the higher expression of *Nnat* should correspond with lower expression of *F2r*. This is what we observe (Figure 4). If this relationship is true, we should see persistent co-expression of *Nnat* and *F2r* across genetic backgrounds (F_0_, F_1_, F_2_), which we do (Figure 4). This supports a biologically meaningful relationship between *Nnat* and *F2r*. Our model further predicts that the DE genes should more closely affect phenotype (Pierce et al., 2014; Shan et al., 2019; Lutz and Hokanson, 2015). In the case of *Nnat* and *F2r*, we expect *F2r* to more strongly associate with basal glucose levels than *Nnat*, which we observe (Figure 4).

There is a clear relationship between *Nnat* and *F2r* in adipogenesis, but the specifics of how this relationship extends to glucose homeostasis are unclear. One possibility is that by altering SERCA function, *Nnat* affects not only the formation of new adipocytes, but also the beiging of adipocytes. The SERCA channel is uncoupled in beige adipocytes as part of a UCP1-independent form of non-shivering thermogenesis (Ikeda and Yamada, 2020). Non-shivering thermogenesis consumes a significant amount of energy, thereby altering glucose homeostasis (Carson et al., 2020). This hypothesis links these genes to physiological processes that are consistent with evolutionary hypotheses about the prevalence of parent-of-origin effects. *Nnat* and *F2r* are members of a putative network we identified that is coordinated by interactions between ASE and DE genes. From the literature, we found that the genes in this epistatic network function in key steps in a pathway regulating differentiation and survival of adipocytes in response to nutritional environment (Figure 3B). Specifically, there is evidence that it plays a critical role in the induction of adipogenesis. This alone demonstrates how parent-of-origin effects can move through networks along molecular pathways. Beyond proof-of-principle this network provides a clue to the puzzle of the prevalence of parent-of-origin effects.

The constituents of this single network appear to play vastly different physiological roles depending on the tissue. In white adipose the network appears to play some role in balancing proliferation, differentiation, and apoptosis as we describe above. In pancreatic ß-cells, members of this network affect insulin secretion (Millership et al., 2018). In bone, members of this network affect the balance of cartilage/bone growth and reabsorption. These three physiological processes may at first seem unrelated, but they share one key commonality – they are jointly critical to growth. This is consistent with the sexual conflict hypothesis attributed to parent-of-origin effects (Patten et al., 2014; Babak et al., 2015). The of size of progeny in placental mammals can have opposing fitness consequences for mothers/ maternal relatives and fathers/ paternal relatives. The fitness of fathers and paternal relatives, particularly in the case of multi-paternity litters, is improved with larger progeny (Mochizuki et al., 1996; Babak et al., 2015; Fowden and Moore, 2012; Patten et al., 2014; Wilkins and Haig, 2003; Haig, 1997). This comes at a fitness disadvantage to the mother. The fitness of mothers is improved by progeny of a manageable size, allowing her to produce multiple litters.

According to this model, imprinting evolved in part to allow one parental lineage to hijack parts of a nutritional environment response pathway driving growth in a direction favorable to maximize the fitness of that lineage. Key processes in such a pathway driving growth would include the secretion of growth factors, construction of cartilage and bone, and the accumulation of energy stores. We present a network that appears to play a role in all three processes. If the sexual conflict hypothesis is true, then the most parsimonious place for imprinting to evolve would be in key regulatory points that affect as many aspects of growth as possible. This is consistent with the network we identified, a single pathway affecting many aspects of growth. This hints at the possibility that parent-of-origin effects are common because of the multi-purpose nature of the pathways in which genomic imprinting manifests and parent-of-origin effects propagate.

By leveraging the reciprocal F_1_ hybrids, we are able to integrate parent-of-origin-dependent allele specific expression and parent-of-origin-dependent differential expression with F_16_ phenotypes. By doing so, we identify plausible candidates for functional validation and describe discrete molecular networks that may contribute to the observed parent-of-origin effects on metabolic phenotypes. The genes and interactions we present here represent a set of actionable interacting candidates that can be probed to further identify the machinery driving these phenomena and make predictions informed by genomic sequence. The frameworks we have developed account for the genetic, epigenetic, and environmental components underlying these parent-of-origin effects, thereby improving our ability to predict complex phenotypes from genomic sequence. We focused on metabolic phenotypes in this study, but the patterns we identified may translate to other complex traits where parent-of-origin effects have been implicated.

## Materials and methods

**Key resources table:** 

Reagent type (species) or resource	Designation	Source or reference	Identifiers	Additional information
Other	High fat diet	Teklad	TD88137	42% kcal from fat
Other	Low-fat diet	Research Diets	D12284	15% kcal from fat
Commercial assay, kit	RNeasy Lipid Tissue Kit	QIAgen	74,804	
Commercial assay, kit	RiboZero kit	Illumina	20040529	
Commercial assay, kit	DNA 1000LabChip	Agilent	5067–1504	
Commercial assay, kit	High-Capacity cDNA Reverse Transcription Kit	Thermo Fisher	4368814	
Sequence-based reagent	*Nnat* forward primer	This paper		Detailed information is found in the methods section
Sequence-based reagent	*Nnat* reverse primer	This paper		Detailed information is found in the methods section
Sequence-based reagent	*F2r* forward primer	This paper		Detailed information is found in the methods section
Sequence-based reagent	*F2r* reverse primer	This paper		Detailed information is found in the methods section
Sequence-based reagent	*L32* forward primer	This paper		Detailed information is found in the methods section
Sequence-based reagent	*L32* reverse primer	This paper		Detailed information is found in the methods section
Software, algorithm	R	R	3.6.1	
Software, algorithm	STAR	STAR	DOI: 10.1093/bioinformatics/bts635	
Software, algorithm	FASTQC	FASTQC	other	https://www.bioinformatics.babraham.ac.uk/projects/fastqc/
Software, algorithm	EdgeR	CRAN	DOI: 10.1093/bioinformatics/btp616	
Software, algorithm	WEB-based Gene Set Analysis Toolkit	WEB-based Gene Set Analysis Toolkit	DOI: 10.1093/nar/gkz401	
Software, algorithm	Seurat	Seurat	DOI: 10.1038/nbt.3192	
Strain, strain background (Mus musculius)	SM/J	The Jackson Laboratory	000687	
Strain, strain background (*Mus musculus*)	LG/J	The Jackson Laboratory	000675	

### Mouse husbandry and phenotyping

LG/J and SM/J founders (F_0_) were obtained from The Jackson Laboratory (Bar Harbor, ME). F_1_ reciprocal cross animals were generated by mating LG/J mothers with SM/J fathers (LxS) and the inverse (SxL). F_2_ intercrossed animals were generated by mating LxS mothers with SxL fathers and the inverse. After weaning at 21 days, animals were separated into sex-specific cages of 3–5 animals per cage and randomly placed on high-fat (42% kcal from fat; Teklad TD88137) or low-fat (15% kcal from fat; Research Diets D12284) isocaloric diets. Feeding was ad libitum. There were 96 experimental F_1_ animals in total, with 48 animals for each cross (LxS and SxL). Within each cross, there were 24 high-fat-fed animals (12 males; 12 females) and 24 low-fat-fed animals (12 males; 12 females). The F_2_ animals were generated for a different study, following the same weaning protocol and diets, and we used data from the high fat-fed females (n = 14) for validation in the the current study (Carson et al., 2020). Additionally, we used data generated from founder F_0_ (LG/J (n = 6) and SM/J (6)) high fat-fed female animals, also generated for a different study and subjected to the same weaning protocol and diets (Carson et al., 2020). The barrier animal facilities at WUSM follow a 12/12 hr light/dark schedule, all water is autoclaved and changed weekly, and all cages are changed weekly.

All animals were weighed weekly from three weeks of age until sacrifice. At 19 weeks of age, body composition was determined by MRI and a glucose tolerance test was performed after a 4 hr fast. At 20 weeks of age, animals were given an overdose of sodium pentobarbital after a 4 hr fast and blood was collected via cardiac puncture. Euthanasia was achieved by cardiac perfusion with phosphate-buffered saline. After cardiac perfusion, the reproductive fat pad was harvested, flash frozen in liquid nitrogen, and stored at –80 °C.

### Study design

The weaning, phenotyping protocols, and diets were chosen to reproduce the protocols and diets used in studies of the F16 Advanced Intercross of the LG/J x SM/J inbred mouse lines that were used in previously published mapping studies (Cheverud et al., 2011; Lawson et al., 2011a; Lawson et al., 2011b, Lawson et al., 2010). The experimental unit for the current study is the individual mouse. For the RNA sequencing, a single animal was randomly chosen from each cage using a random number generator in R. All other animals served as biological replicates. Mice from multiple cages representing different crosses, generations, diets, and sexes, were necropsied at the same time to avoid batch effects. Library prep and RNA sequencing was performed blinded by the WUSM Genome Technology and Access Center.

### Genomes and annotations

LG/J and SM/J indels and SNVs were leveraged to construct strain-specific genomes using the GRC38.72-mm10 reference as a template (Nikolskiy et al., 2015). This was done by replacing reference bases with alternative (LG/J | SM/J) bases using custom python scripts. Ensembl R72 annotations were adjusted for indel-induced indexing differences for both genomes.

### RNA sequencing

Total RNA was isolated from adipose tissue using the RNeasy Lipid Tissue Kit (QIAgen) (n = 32, 4 animals per sex/diet/cross cohort). RNA concentration was measured via NanoDrop and RNA quality/integrity was assessed with a BioAnalyzer (Agilent). RNA-Seq libraries were constructed using the RiboZero kit (Illumina) from total RNA samples with RIN scores > 8.0. Libraries were checked for quality and concentration using the DNA 1000LabChip assay (Agilent) and quantitative PCR, according to manufacturer’s protocol. Libraries were sequenced at 2 × 100 paired end reads on an Illumina HiSeq 4,000. After sequencing, reads were de-multiplexed and assigned to individual samples. RNAseq data are available through the NCBI-SRA, accession: PRJNA753198.

### Library complexity

Complexity was measured by fitting a beta-binomial distribution to the distribution of L_bias_ values using the VGAM package (Yee, 2010). The shape parameters (α, β) of beta-binomial distributions were estimated and used to calculate dispersion (ρ). Dispersion values less than 0.05 indicate our libraries are sufficiently complex (Figure 2—figure supplement 1).

$$\rho_{s}=\frac{1}{1+\alpha_{s}+\beta_{s}}$$

One library was found to have insufficient complexity and was removed from the analyses.

### Allele-specific expression

FASTQ files were filtered to remove low quality reads and aligned against both LG/J and SM/J pseudo-genomes simultaneously using STAR with multimapping disallowed (Dobin et al., 2013). Read counts were normalized via upper quartile normalization and a minimum normalized read depth of 20 was required. Alignment summaries are provided in Supplementary file 8 and Figure 2—figure supplement 2.

For each gene in each individual, allelic bias (L_bias_) was calculated as the proportion of total reads for a given gene with the LG/J haplotype. Parent-of-origin-dependent allele-specific expression was detected by ANOVA using one of a number of models in which L_bias_ is responsive to cross and the interaction of cross with some combination of sex and diet:

$$Model\;\left\{ \begin{array}{l} if\;each\;Cross\;context\;has\geq 2\;samples,\;Lbias∼Cross\;\\ if\;each\;Cross:Sex\;context\;has\geq 2\;samples,\;Lbias∼Cross+Cross:Sex\;\\ if\;each\;Cross:Diet\;context\;has\geq 2\;samples,\;\;Lbias∼Cross+Cross:Diet\\ \;if\;each\;context\;has\geq 2\;samples,\;\;Lbias∼Cross+Cross:Sex+Cross:Diet+Cross:Sex:Diet\; \end{array}\right.$$

Accurately estimating the significance of these effects and correcting for multiple tests is challenging for two reasons: (1) the complexity of the many environmental contexts and (2) the correlation of allelic bias within and between imprinted domains breaks assumptions of independence. A permutation approach is an effective way to overcome these challenges. The context data was randomly shuffled for each gene independently and analyses were rerun to generate a stable null distribution of p-values (Figure 2—figure supplement 3). False discovery rates were estimated for a given significance threshold as the proportion of significant tests under the permutated null model relative to significant tests under the real data model. A value of 1 meaning that 100% of tests at a given significance threshold are likely false positives. An FDR ≤ 0.1 was considered significant (Supplementary file 1, Figure 2—figure supplement 4).

To determine the parental direction and size of expression biases, a parent-of-origin effect score was calculated as the difference in mean L_bias_ between reciprocal crosses (LxS or SxL). Parent-of-origin effect scores range from completely maternally expressed (–1), to biallelic (0), to completely paternally expressed ( + 1). Parent-of-origin effect score thresholds were calculated from a critical value of *α* = 0.01, determined from a null distribution created by permutation Genes with significant allele-specific expression and parent-of-origin scores beyond the critical value were considered to have significant parent-of-origin-dependent allele-specific expression (Figure 2—figure supplement 5).

### Differential expression

Differential expression by reciprocal cross was determined by first aligning reads against the LG/J and SM/J genomes simultaneously with multimapping permitted. Reads were normalized by Trimmed mean of M-values (TMM) normalization, which estimates scale factors among samples to allow for differences in RNA composition (Robinson and Oshlack, 2010). A minimum normalized read count of 10 was required. Generalized linear models accounting for diet, sex, and diet-by-sex were fit in EdgeR (Robinson et al., 2010). Differential expression was detected by a likelihood ratio test. Significance was determined for five models for each gene:

1.Expression~Cross

2.Expression~Cross:Sex

3.Expression~Cross:Diet

4.Expression~Cross:Sex:Diet

5.Expression~Cross+Cross:Sex+Cross:Diet+Cross:Sex:Diet

Multiple test corrections were performed by implementing the ‘qvalue’ R package to estimate false discovery rates (Figure 2—figure supplement 6). Genes with a FDR of ≤0.1 and a $\left|foldchange\right|\geq 1.5$ were considered significantly differentially expressed by reciprocal cross (Figure 2—figure supplement 7 and Supplementary file 2).

### Gene-gene interactions

Networks were constructed in each tissue by pairing genes showing parent-of-origin-dependent allele-specific expression with biallelic genes that are differentially expressed by cross. Pairs were predicted by modeling the expression of biallelic genes as a function of parent-of-origin-dependent allele-specific expression, L_bias_, sex, diet, and sex-by-diet. The strength of a prediction was measured through model fit, which was estimated as a mean test error with 10-fold cross-validation employed to prevent overfitting. Significance was estimated by likelihood ratio test using a chi-square distribution. Given the complexity of contexts, false discovery rates were determined by permuting the context and expression data to generate a stable null-distribution of p-value (Figure 2—figure supplement 8) Null distribution stability was evaluated by calculating the critical value for alpha = 0.05 at each genome wide iteration. The standard deviation of critical values was calculated after each iteration for the last 5 iterations. Genome-wide shuffling was done 500 times, with 759 independent randomized tests per iteration, meaning the stable null model is composed of 379,500 randomized observations. Using the null model, the ‘qvalue’ package estimated a $\hat{\pi_{\text{0}}}$ . This estimate was then used to estimate false discovery rates in the real data. MTE score thresholds were calculated from a critical value of *α* = 0.01, determined from a null distribution created by permutation (Figure 2—figure supplement 9). Connections with an FDR ≤ 0.1 (Supplementary file 9) and MTE below the critical value were considered significant (Figure 2—figure supplement 10).

### Functional enrichment analysis

Functional enrichment of interacting genes showing parent-of-origin-dependent allele-specific expression with biallelic genes that are differentially expressed by cross was tested by over-representation analysis in the WEB-based Gene Set Analysis Toolkit v2019 (Zhang et al., 2005). We performed analyses of gene ontologies (biological process, cellular component, and molecular function), pathway (KEGG), and phenotype (Mammalian Phenotype Ontology). The list of all unique interacting genes was analyzed against the background of all unique genes expressed in white adipose. A Benjamini-Hochberg FDR-corrected p-value ≤ 0.01 was considered significant (Supplementary file 4).

### Phenotype correlation

In order to identify which phenotypes might be affected by genes in the parent-of-origin effects network, gene expression was correlated with metabolic phenotypes collected for F_1_ animals with the contexts combined. Phenotypes were log transformed when necessary, as determined by Shapiro Wilkes test to approximate normality (Figure 2—figure supplement 11). Additionally, the effects of sex and diet were residualized out leaving only the effect of cross. This was done to mirror later residualizing of phenotypes in the F16 population when testing for epistasis. Multiple test corrections were performed by implementing the ‘qvalue’ R package to estimate false discovery rates (Figure 2—figure supplement 12). The minimum Pearson’s correlation coefficient threshold was set to |0.5|. Connections with an FDR ≤ 0.05 (Supplementary file 5) and MTE below the critical value were considered significant (Figure 2—figure supplement 13).

### Epistasis testing

The F_16_ LxS advanced intercross population, phenotypes, genotypes, genotypic scores, and QTL mapping methods are described elsewhere (Cheverud et al., 2011; Lawson et al., 2011a; Lawson et al., 2011b, Lawson et al., 2010). We tested for epistasis in interacting pairs between genes showing parent-of-origin-dependent allele-specific expression and biallelic genes that are differentially expressed by cross. We selected F_16_ genotyped markers that fall within 1.5 Mb up- and downstream from the geometric center of each gene, defined as the genomic position halfway between the transcription start and stop position of that gene (Supplementary file 10). For every F_16_ animal, an ‘imprinting score’ was assigned to each marker based on that animal’s genotypic values (LL = 0, LS = 1, SL = –1, SS = 0; maternal allele is depicted first). Non-normally distributed phenotypes (as evaluated by a Shapiro-Wilk test) were log_10_-transformed to approximate normality (Figure 3—figure supplement 1). Because of the number of epistasis tests performed and the number of contexts represented in the data, we removed the effects of sex, diet and their interaction from each F_16_ phenotype with a covariate screen. We tested for epistasis on the residualized data using the following generalized linear model:

$$R_{pheno}\sim {BDE}_{IMP}+{ASE}_{IMP}+{BDE}_{IMP}:{ASE}_{IMP}$$

where *R_pheno_* is the residual phenotype, *BDE_IMP_* is the imprinted genotypic score for the biallelic gene that is differentially expressed by cross, *ASE_IMP_* is the imprinted genotypic score for the gene showing parent-of-origin-dependent allele-specific expression bias, and *BDE_IMP_:ASE_IMP_* is the interaction between the two genes’ imprinted genotypic score. We employed a permutation approach to accurately estimate significance given the linkage of proximal markers. Imprinted genotypic values were randomly shuffled to generate a stable null model of p-values (Figure 3—figure supplement 2). False discovery rates were estimated for a given significance threshold as the proportion of significant tests under the permutated null model relative to significant tests under the real data model (Figure 3—figure supplement 3). An FDR ≤ 0.1 was considered significant. Epistasis was considered significant if the *BDE_IMP_: ASE_IMP_* interaction term met the significance threshold (Supplementary file 6).

### Validation of Nnat and F2r expression patterns

Expression patterns of *Nnat* and *F2r* in white adipose were validated by qRT-PCR in high-fat-fed female LG/J and SM/J mice and in biological replicates of high-fat-fed female F_1_ reciprocal cross animals (n = 6 LG/J homozygotes, n = 10 LxS and 10 SxL reciprocal heterozygotes, n = 6 SM/J homozygotes). Total RNA was extracted from adipose samples using the Qiagen RNeasy Lipid Kit. High-Capacity cDNA Reverse Transcription Kit (Thermo Fisher) was used for reverse transcription. Quantitative RT-PCR was performed with an Applied Biosystems (USA) QuantStudio 6 Flex instrument using SYBR Green reagent. Results were normalized to *L32* expression using the ΔΔCt method. *Nnat* forward primer – CTACCCCAAGAGCTCCCTTT and reverse primer – CAGCTTCTGCAGGGAGTACC. *F2r* forward primer – TGAACCCCCGCTCATTCTTTC and reverse primer – CCAGCAGGACGCTTTCATTTTT. *L32* forward primer – TCCACAATGTCAAGGAGCTG and reverse primer – GGGATTGGTGACTCTGATGG. Data points were considered outliers if they led to violation of normality assumptions or were considered outliers by box and whisker plots. ANOVA was used to estimate significance of differential expression by cross (1), paternal allele identity (2), mitochondrial ancestry (3).

$$1.\;Expression\;∼\;Cross\;\in \;\left\{ \begin{array}{l} LL,\;0\\ \;LS,\;-1\\ \;SL,\;1\\ SS,\;0 \end{array}\right.$$

$$2.\;Expression\;∼\;Paternal\;Allele\;\in \;\left\{ \begin{array}{l} \;LL,\;0\\ \;LS,\;1\\ SL,\;0\;\\ SS,1 \end{array}\right.$$

$$3.\;Expression\;∼\;Mitochondrial\;ancestry\;\in \;\left\{ \begin{array}{l} \;LxS\;x\;SxL,\;0\\ \;SxL\;x\;LxS,\;1 \end{array}\right.$$

Expression patterns were also validated by qRT-PCR in high fat-fed female F_2_ animals (n = 14). Co-expression was determined by fitting a general linear model and estimating significance using the Wald test approximation of the LR test. Correlation with basal glucose was determined by fitting a general linear model and estimating significance using the Wald test approximation of the LR test. Pearson’s correlation coefficients were calculated for each gene with basal glucose. To test whether patterns in these correlations was significantly different between F_0_/F_1_ and F_2_ populations, bootstrapping was used to calculate 90% confidence intervals for the Pearson’s correlation coefficients. 5,000 iterations were run with 10 individuals randomly selected with replacement. scRNA analysis of Nnat and F2r scRNAseq data was downloaded from SRA: SRP145475 (Burl et al., 2018). Data were processed and aligned to the C57BL/6 J reference (mm10) using Cell Ranger (Zheng et al., 2017). Analysis and cell quality control was performed using the Seurat (3.2.2)(Stuart et al., 2019) package in R (3.6.1)(R Development Core Team, 2013). Cell quality was controlled using three metrics (Luecken and Theis, 2019): (1) number of features, (2) number of counts, (3) covariation of features and counts. High quality cells were required to have between 500 and 3000 features and read counts between 1000 and 30,000. As sequencing is a process of random sampling, the number of features and the number of counts should covary. This relationship was fit to a generalized additive model. Deviation from this relationship (residuals) were computed for each cell. High-quality cells were required to have a residual within 3 standard deviations of the mean residual of all cells (Figure 5—figure supplement 3).

Seurat normalization with a scale factor of 10,000 was performed. Dimensionality reduction (UMAP) was performed (dims = 1:10, resolution = 0.15). Resolution was chosen using the clustree (0.4.3) package (Zappia and Oshlack, 2018). A range of resolutions from 0.06 to 0.18 were tested, and the highest resolution with stable clustering was chosen (Figure 5—figure supplement 4). Cell type markers were identified by differential expression analysis using the ‘MAST’ hurdle-model test (Finak et al., 2015). Genes overexpressed in a given cell type relative to all other cell types were considered cell type ‘markers’. Cell type identity was assigned using previously reported markers for this data set (Figure 5—figure supplement 1).

Cells along the adipogenic trajectory were subset and subjected to dimensionality reduction (UMAP, dims = 1:10, resolution = 0.17). A range of resolutions from 0.01 to 0.25 were tested. Using *Adipoq* as a marker of differentiation, we sought to identify the set of clusters that would best encapsulate the stages of differentiation. To this end for every level of resolution we calculated the mean count variance ($\bar{C_{\sigma }}$). This is done by calculating the standard deviation (σ) of *Adipoq* expression (E) within each cluster (G), referred to as the count variance ($C_{\sigma }$). Cells with no expression of *Adipoq* were excluded. The mean of count variances for all clusters is calculated. This process is similar to k-means clustering, where the goal is to find that parameters which minimize the within group variation.

$$Count\;Variance=\;\frac{\sum_{G=1}^{n}\sigma \left(E_{G}\right)}{n}$$

We also calculated the percent expressing variance ($\bar{P_{\sigma }}$). This was taken as the mean of the standard deviation in the percent of cells expressing *Adipoq*.

$$Percent\;Expressing\;Variance=\;\frac{\sum_{G=1}^{n}\sigma \left(\%E>0_{G}\right)}{n}$$

The resolution 0.17 was chosen as the lowest resolution where variation is minimized and no longer significantly changes (Figure 5—figure supplement 5). Using *Adipoq* as a marker of adipogenesis, clusters 1 and 2 were identified as pre- and post-differentiated cells, respectively. Differential expression was analyzed using the ‘MAST’ test. Expression was compared between clusters 1 and 2 only. Multiple tests correction was performed using the Bonferroni method. We required changes in expression to show either a sufficiently large fold change ($\left|\log_{2}FoldChange\right|\geq 0.3$) *OR* a sufficiently large change in the percent of cells expressing the gene in question (pct.∆≥0.4). The change in percent of cells expressing a gene was calculated as the difference in percent of cells expressing the gene between the clusters and scaled by dividing by the larger percentage.

$$pct.∆=\frac{pct.2-pct.1}{max(pct.1,pct.2)}$$

Source code is available at https://github.com/LawsonLab-WUSM/POE_Epistasis, (copy archived at swh:1:rev:b39046ce35f53e0c3f15bcdefa122c274aee48b7, Lawson, 2019).

## Data Availability

Sequencing data are available through the NCBI-SRA under accession code PRJNA753198. The following dataset was generated: LawsonHA 2021LG/J and SM/J hybrid RNAseqNCBI BioProjectPRJNA753198 The following previously published dataset was used: BurlRB 2018Single-cell RNA-sequencing of white adipose tissue stromal cells during CL-induced adipogenesisEuropean Nucleotide ArchivePRJNA470640

## Additional files

Supplementary file 1. Allele-specific expression.

Supplementary file 2. Biallelic genes differentially expressed by cross.

Supplementary file 3. Networks of genes showing parent-of-origin allele-specific expression interacting with biallelic genes that are differentially expressed by cross.

Supplementary file 4. Over-representation input/output.

Supplementary file 5. F_1_ Phenotype correlation.

Supplementary file 6. Epistasis results.

Supplementary file 7. Cell cluster markers.

Supplementary file 8. Alignment summaries.

Supplementary file 9. List of imprinted genes queried.

Supplementary file 10. Genome annotations.

Transparent reporting form

## References

[bib1] Akune T, Ohba S, Kamekura S, Yamaguchi M, Chung U-I, Kubota N, Terauchi Y, Harada Y, Azuma Y, Nakamura K, Kadowaki T, Kawaguchi H (2004). PPARgamma insufficiency enhances osteogenesis through osteoblast formation from bone marrow progenitors. The Journal of Clinical Investigation.

[bib2] Ali AA, Weinstein RS, Stewart SA, Parfitt AM, Manolagas SC, Jilka RL (2005). Rosiglitazone causes bone loss in mice by suppressing osteoblast differentiation and bone formation. Endocrinology.

[bib3] An JJ, Han DH, Kim DM, Kim SH, Rhee Y, Lee EJ, Lim SK (2007). Expression and regulation of osteoprotegerin in adipose tissue. Yonsei Medical Journal.

[bib4] Aubin JE (1992). Osteoclast adhesion and resorption: the role of podosomes. Journal of Bone and Mineral Research.

[bib5] Babak T, DeVeale B, Tsang EK, Zhou Y, Li X, Smith KS, Kukurba KR, Zhang R, Li JB, van der Kooy D, Montgomery SB, Fraser HB (2015). Genetic conflict reflected in tissue-specific maps of genomic imprinting in human and mouse. Nature Genetics.

[bib6] Berridge MJ (2016). The Inositol Trisphosphate/Calcium Signaling Pathway in Health and Disease. Physiological Reviews.

[bib7] Bigdeli N, de Peppo GM, Lennerås M, Sjövall P, Lindahl A, Hyllner J, Karlsson C (2010). Superior osteogenic capacity of human embryonic stem cells adapted to matrix-free growth compared to human mesenchymal stem cells. Tissue Engineering. Part A.

[bib8] Burg JS, Espenshade PJ (2011). Regulation of HMG-CoA reductase in mammals and yeast. Progress in Lipid Research.

[bib9] Burl RB, Ramseyer VD, Rondini EA, Pique-Regi R, Lee YH, Granneman JG (2018). Deconstructing Adipogenesis Induced by β3-Adrenergic Receptor Activation with Single-Cell Expression Profiling. Cell Metabolism.

[bib10] Carson C, Macias-Velasco JF, Gunawardana S, Miranda MA, Oyama S, St Pierre CL, Schmidt H, Wayhart JP, Lawson HA (2020). Brown Adipose Expansion and Remission of Glycemic Dysfunction in Obese SM/J Mice. Cell Reports.

[bib11] Cheverud JM, Lawson HA, Fawcett GL, Wang B, Pletscher LS, R Fox A, Maxwell TJ, Ehrich TH, Kenney-Hunt JP, Wolf JB, Semenkovich CF (2011). Diet-dependent genetic and genomic imprinting effects on obesity in mice. Obesity (Silver Spring, Md.).

[bib12] Cho SW, Yang J-Y, Her SJ, Choi HJ, Jung JY, Sun HJ, An JH, Cho HY, Kim SW, Park KS, Kim SY, Baek W-Y, Kim J-E, Yim M, Shin CS (2011). Osteoblast-targeted overexpression of PPARγ inhibited bone mass gain in male mice and accelerated ovariectomy-induced bone loss in female mice. Journal of Bone and Mineral Research.

[bib13] Dobin A, Davis CA, Schlesinger F, Drenkow J, Zaleski C, Jha S, Batut P, Chaisson M, Gingeras TR (2013). STAR: ultrafast universal RNA-seq aligner. Bioinformatics (Oxford, England).

[bib14] Erol M, Bostan Gayret O, Tekin Nacaroglu H, Yigit O, Zengi O, Salih Akkurt M, Tasdemir M (2016). Association of Osteoprotegerin with Obesity, Insulin Resistance and Non-Alcoholic Fatty Liver Disease in Children. Iranian Red Crescent Medical Journal.

[bib15] Finak G, McDavid A, Yajima M, Deng J, Gersuk V, Shalek AK, Slichter CK, Miller HW, McElrath MJ, Prlic M, Linsley PS, Gottardo R (2015). MAST: a flexible statistical framework for assessing transcriptional changes and characterizing heterogeneity in single-cell RNA sequencing data. Genome Biology.

[bib16] Font-Clos F, Zapperi S, La Porta CAM (2017). Integrative analysis of pathway deregulation in obesity. NPJ Systems Biology and Applications.

[bib17] Fowden AL, Moore T (2012). Maternal-fetal resource allocation: co-operation and conflict. Placenta.

[bib18] Franck N, Gummesson A, Jernås M, Glad C, Svensson PA, Guillot G, Rudemo M, Nyström FH, Carlsson LMS, Olsson B (2011). Identification of adipocyte genes regulated by caloric intake. The Journal of Clinical Endocrinology and Metabolism.

[bib19] Hager R, Cheverud JM, Wolf JB (2008). Maternal effects as the cause of parent-of-origin effects that mimic genomic imprinting. Genetics.

[bib20] Haig D (1997). Parental antagonism, relatedness asymmetries, and genomic imprinting. Proceedings. Biological Sciences.

[bib21] Holecki M, Zahorska-Markiewicz B, Janowska J, Nieszporek T, Wojaczyńska-Stanek K, Zak-Gołab A, Wiecek A (2007). The influence of weight loss on serum osteoprotegerin concentration in obese perimenopausal women. Obesity (Silver Spring, Md.).

[bib22] Ikeda K, Yamada T (2020). UCP1 Dependent and Independent Thermogenesis in Brown and Beige Adipocytes. Frontiers in Endocrinology.

[bib23] Imoto-Tsubakimoto H, Takahashi T, Ueyama T, Ogata T, Adachi A, Nakanishi N, Mizushima K, Naito Y, Matsubara H (2013). Serglycin is a novel adipocytokine highly expressed in epicardial adipose tissue. Biochemical and Biophysical Research Communications.

[bib24] Jirtle RL (2012). Geneimprint.

[bib25] Jo Y, Debose-Boyd RA (2010). Control of cholesterol synthesis through regulated ER-associated degradation of HMG CoA reductase. Critical Reviews in Biochemistry and Molecular Biology.

[bib26] Kang JW, Choi Y, Park JH, Kim JS, Park KD, Baek DH, Seong SK, Choi KS, Lim SY, Kim HS (2008). The effects of cyclin-dependent kinase inhibitors on adipogenic differentiation of human mesenchymal stem cells. Biochemical and Biophysical Research Communications.

[bib27] Kichaev G, Bhatia G, Loh PR, Gazal S, Burch K, Freund MK, Schoech A, Pasaniuc B, Price AL (2019). Leveraging Polygenic Functional Enrichment to Improve GWAS Power. American Journal of Human Genetics.

[bib28] Kong A, Steinthorsdottir V, Masson G, Thorleifsson G, Sulem P, Besenbacher S, Jonasdottir A, Sigurdsson A, Kristinsson KT, Jonasdottir A, Frigge ML, Gylfason A, Olason PI, Gudjonsson SA, Sverrisson S, Stacey SN, Sigurgeirsson B, Benediktsdottir KR, Sigurdsson H, Jonsson T, Benediktsson R, Olafsson JH, Johannsson OT, Hreidarsson AB, Sigurdsson G, Ferguson-Smith AC, Gudbjartsson DF, Thorsteinsdottir U, Stefansson K, DIAGRAM Consortium (2009). Parental origin of sequence variants associated with complex diseases. Nature.

[bib29] Lawson HA, Zelle KM, Fawcett GL, Wang B, Pletscher LS, Maxwell TJ, Ehrich TH, Kenney-Hunt JP, Wolf JB, Semenkovich CF, Cheverud JM (2010). Genetic, epigenetic, and gene-by-diet interaction effects underlie variation in serum lipids in a LG/JxSM/J murine model. Journal of Lipid Research.

[bib30] Lawson HA, Cady JE, Partridge C, Wolf JB, Semenkovich CF, Cheverud JM (2011a). Genetic effects at pleiotropic loci are context-dependent with consequences for the maintenance of genetic variation in populations. PLOS Genetics.

[bib31] Lawson HA, Lee A, Fawcett GL, Wang B, Pletscher LS, Maxwell TJ, Ehrich TH, Kenney-Hunt JP, Wolf JB, Semenkovich CF, Cheverud JM (2011b). The importance of context to the genetic architecture of diabetes-related traits is revealed in a genome-wide scan of a LG/J × SM/J murine model. Mammalian Genome.

[bib32] Lawson HA, Cheverud JM, Wolf JB (2013). Genomic imprinting and parent-of-origin effects on complex traits. Nature Reviews. Genetics.

[bib33] Lawson HA (2019). Software Heritage.

[bib34] Lemire JM, Chan CK, Bressler S, Miller J, LeBaron RG, Wight TN (2007). Interleukin-1beta selectively decreases the synthesis of versican by arterial smooth muscle cells. Journal of Cellular Biochemistry.

[bib35] Li X, Thomason PA, Withers DJ, Scott J (2010). Bio-informatics analysis of a gene co-expression module in adipose tissue containing the diet-responsive gene Nnat. BMC Systems Biology.

[bib36] Lin HH, Bell E, Uwanogho D, Perfect LW, Noristani H, Bates TJD, Snetkov V, Price J, Sun YM (2010). Neuronatin promotes neural lineage in ESCs via Ca(2+) signaling. Stem Cells (Dayton, Ohio).

[bib37] Luecken MD, Theis FJ (2019). Current best practices in single-cell RNA-seq analysis: a tutorial. Molecular Systems Biology.

[bib38] Lutz SM, Hokanson JE (2015). Mediation analysis in genome-wide association studies: current perspectives. Open Access Bioinformatics.

[bib39] Matsuo FS, Cavalcanti de Araújo PH, Mota RF, Carvalho AJR, Santos de Queiroz M, Baldo de Almeida B, Ferreira KC, Metzner RJM, Ferrari GD, Alberici LC, Osako MK (2020). RANKL induces beige adipocyte differentiation in preadipocytes. American Journal of Physiology. Endocrinology and Metabolism.

[bib40] McDonald LJ, Mamrack MD (1995). Phosphoinositide hydrolysis by phospholipase C modulated by multivalent cations La(3+), Al(3+), neomycin, polyamines, and melittin. Journal of Lipid Mediators and Cell Signalling.

[bib41] Millership SJ, Da Silva Xavier G, Choudhury AI, Bertazzo S, Chabosseau P, Pedroni SM, Irvine EE, Montoya A, Faull P, Taylor WR, Kerr-Conte J, Pattou F, Ferrer J, Christian M, John RM, Latreille M, Liu M, Rutter GA, Scott J, Withers DJ (2018). Neuronatin regulates pancreatic β cell insulin content and secretion. The Journal of Clinical Investigation.

[bib42] Miranda MA, Carson C, St. Pierre CL, Macias‐Velasco JF, Hughes JW, Kunzmann M, Schmidt H, Wayhart JP, Lawson HA (2020). Spontaneous restoration of functional β‐cell mass in obese SM/J mice. Physiological Reports.

[bib43] Mochizuki A, Takeda Y, Iwasa Y (1996). The evolution of genomic imprinting. Genetics.

[bib44] Mott R, Yuan W, Kaisaki P, Gan X, Cleak J, Edwards A, Baud A, Flint J (2014). The architecture of parent-of-origin effects in mice. Cell.

[bib45] Mozaffari SV, DeCara JM, Shah SJ, Sidore C, Fiorillo E, Cucca F, Lang RM, Nicolae DL, Ober C (2019). Parent-of-origin effects on quantitative phenotypes in a large Hutterite pedigree. Communications Biology.

[bib46] Nakagawa N, Kinosaki M, Yamaguchi K, Shima N, Yasuda H, Yano K, Morinaga T, Higashio K (1998). RANK is the essential signaling receptor for osteoclast differentiation factor in osteoclastogenesis. Biochemical and Biophysical Research Communications.

[bib47] Nakamura Y, Hamada Y, Fujiwara T, Enomoto H, Hiroe T, Tanaka S, Nose M, Nakahara M, Yoshida N, Takenawa T, Fukami K (2005). Phospholipase C-delta1 and -delta3 are essential in the trophoblast for placental development. Molecular and Cellular Biology.

[bib48] Nikolskiy I, Conrad DF, Chun S, Fay JC, Cheverud JM, Lawson HA (2015). Using whole-genome sequences of the LG/J and SM/J inbred mouse strains to prioritize quantitative trait genes and nucleotides. BMC Genomics.

[bib49] Parker-Katiraee L, Carson AR, Yamada T, Arnaud P, Feil R, Abu-Amero SN, Moore GE, Kaneda M, Perry GH, Stone AC, Lee C, Meguro-Horike M, Sasaki H, Kobayashi K, Nakabayashi K, Scherer SW (2007). Identification of the imprinted KLF14 transcription factor undergoing human-specific accelerated evolution. PLOS Genetics.

[bib50] Patten MM, Ross L, Curley JP, Queller DC, Bonduriansky R, Wolf JB (2014). The evolution of genomic imprinting: theories, predictions and empirical tests. Heredity.

[bib51] Pelled D, Lloyd-Evans E, Riebeling C, Jeyakumar M, Platt FM, Futerman AH (2003). Inhibition of calcium uptake via the sarco/endoplasmic reticulum Ca2+-ATPase in a mouse model of Sandhoff disease and prevention by treatment with N-butyldeoxynojirimycin. The Journal of Biological Chemistry.

[bib52] Pierce BL, Tong L, Chen LS, Rahaman R, Argos M, Jasmine F, Roy S, Paul-Brutus R, Westra HJ, Franke L, Esko T, Zaman R, Islam T, Rahman M, Baron JA, Kibriya MG, Ahsan H (2014). Mediation analysis demonstrates that trans-eQTLs are often explained by cis-mediation: A genome-wide analysis among 1,800 South Asians. PLOS Genetics.

[bib53] Prockop DJ (1997). Marrow stromal cells as stem cells for nonhematopoietic tissues. Science (New York, N.Y.).

[bib54] R Development Core Team (2013). http://www.r-project.org.

[bib55] Renner SW, Walker LM, Forsberg LJ, Sexton JZ, Brenman JE (2017). Carbonic anhydrase III (Car3) is not required for fatty acid synthesis and does not protect against high-fat diet induced obesity in mice. PLOS ONE.

[bib56] Robinson MD, McCarthy DJ, Smyth GK (2010). edgeR: a Bioconductor package for differential expression analysis of digital gene expression data. Bioinformatics (Oxford, England).

[bib57] Robinson MD, Oshlack A (2010). A scaling normalization method for differential expression analysis of RNA-seq data. Genome Biology.

[bib58] Rodríguez JP, Astudillo P, Ríos S, Pino AM (2008). Involvement of adipogenic potential of human bone marrow mesenchymal stem cells (MSCs) in osteoporosis. Current Stem Cell Research & Therapy.

[bib59] Rosen CJ, Bouxsein ML (2006). Mechanisms of disease: is osteoporosis the obesity of bone?. Nature Clinical Practice. Rheumatology.

[bib60] Schick BP, Gradowski JF, San Antonio JD (2001). Synthesis, secretion, and subcellular localization of serglycin proteoglycan in human endothelial cells. Blood.

[bib61] Shan N, Wang Z, Hou L (2019). Identification of trans-eQTLs using mediation analysis with multiple mediators. BMC Bioinformatics.

[bib62] Small KS, Hedman AK, Grundberg E, Nica AC, Thorleifsson G, Kong A, Thorsteindottir U, Shin S-Y, Richards HB, Soranzo N, Ahmadi KR, Lindgren CM, Stefansson K, Dermitzakis ET, Deloukas P, Spector TD, McCarthy MI, GIANT Consortium, MAGIC Investigators, DIAGRAM Consortium, MuTHER Consortium (2011). Identification of an imprinted master trans regulator at the KLF14 locus related to multiple metabolic phenotypes. Nature Genetics.

[bib63] Soh UJK, Dores MR, Chen B, Trejo J (2010). Signal transduction by protease-activated receptors. British Journal of Pharmacology.

[bib64] Stanton LW, Ponte PA, Coleman RT, Snyder MA (1991). Expression of CA III in rodent models of obesity. Molecular Endocrinology (Baltimore, Md.).

[bib65] Stuart T, Butler A, Hoffman P, Hafemeister C, Papalexi E, Mauck WM, Hao Y, Stoeckius M, Smibert P, Satija R (2019). Comprehensive Integration of Single-Cell Data. Cell.

[bib66] Teslovich TM, Musunuru K, Smith AV, Edmondson AC, Stylianou IM, Koseki M, Pirruccello JP, Ripatti S, Chasman DI, Willer CJ, Johansen CT, Fouchier SW, Isaacs A, Peloso GM, Barbalic M, Ricketts SL, Bis JC, Aulchenko YS, Thorleifsson G, Feitosa MF, Chambers J, Orho-Melander M, Johnson T, Li X, Guo X, Li M, Shin Cho Y, Jin Go M, Jin Kim Y, Lee JY, Park T, Kim K, Sim X, Twee-Hee Ong R, Croteau-Chonka DC, Lange LA, Smith JD, Song K, Hua Zhao J, Yuan X, Luan J, Lamina C, Ziegler A, Zhang W, Zee RYL, Wright AF, Witteman JCM, Wilson JF, Willemsen G, Wichmann HE, Whitfield JB, Waterworth DM, Wareham NJ, Waeber G, Vollenweider P, Voight BF, Vitart V, Uitterlinden AG, Uda M, Tuomilehto J, Thompson JR, Tanaka T, Surakka I, Stringham HM, Spector TD, Soranzo N, Smit JH, Sinisalo J, Silander K, Sijbrands EJG, Scuteri A, Scott J, Schlessinger D, Sanna S, Salomaa V, Saharinen J, Sabatti C, Ruokonen A, Rudan I, Rose LM, Roberts R, Rieder M, Psaty BM, Pramstaller PP, Pichler I, Perola M, Penninx B, Pedersen NL, Pattaro C, Parker AN, Pare G, Oostra BA, O’Donnell CJ, Nieminen MS, Nickerson DA, Montgomery GW, Meitinger T, McPherson R, McCarthy MI, McArdle W, Masson D, Martin NG, Marroni F, Mangino M, Magnusson PKE, Lucas G, Luben R, Loos RJF, Lokki ML, Lettre G, Langenberg C, Launer LJ, Lakatta EG, Laaksonen R, Kyvik KO, Kronenberg F, König IR, Khaw KT, Kaprio J, Kaplan LM, Johansson A, Jarvelin MR, Janssens A, Ingelsson E, Igl W, Kees Hovingh G, Hottenga JJ, Hofman A, Hicks AA, Hengstenberg C, Heid IM, Hayward C, Havulinna AS, Hastie ND, Harris TB, Haritunians T, Hall AS, Gyllensten U, Guiducci C, Groop LC, Gonzalez E, Gieger C, Freimer NB, Ferrucci L, Erdmann J, Elliott P, Ejebe KG, Döring A, Dominiczak AF, Demissie S, Deloukas P, de Geus EJC, de Faire U, Crawford G, Collins FS, Chen YI, Caulfield MJ, Campbell H, Burtt NP, Bonnycastle LL, Boomsma DI, Boekholdt SM, Bergman RN, Barroso I, Bandinelli S, Ballantyne CM, Assimes TL, Quertermous T, Altshuler D, Seielstad M, Wong TY, Tai ES, Feranil AB, Kuzawa CW, Adair LS, Taylor HA, Borecki IB, Gabriel SB, Wilson JG, Holm H, Thorsteinsdottir U, Gudnason V, Krauss RM, Mohlke KL, Ordovas JM, Munroe PB, Kooner JS, Tall AR, Hegele RA, Kastelein JJP, Schadt EE, Rotter JI, Boerwinkle E, Strachan DP, Mooser V, Stefansson K, Reilly MP, Samani NJ, Schunkert H, Cupples LA, Sandhu MS, Ridker PM, Rader DJ, van Duijn CM, Peltonen L, Abecasis GR, Boehnke M, Kathiresan S (2010). Biological, clinical and population relevance of 95 loci for blood lipids. Nature.

[bib67] Thatcher JD (2010). The inositol trisphosphate (IP3) signal transduction pathway. Science Signaling.

[bib68] Turner RT, Martin SA, Iwaniec UT (2018). Metabolic Coupling Between Bone Marrow Adipose Tissue and Hematopoiesis. Current Osteoporosis Reports.

[bib69] Van de Pette M, Tunster SJ, John RM (2018). Loss of Imprinting of *Cdkn1c* Protects against Age and Diet-Induced Obesity. International Journal of Molecular Sciences.

[bib70] Voight BF, Scott LJ, Steinthorsdottir V, Morris AP, Dina C, Welch RP, Zeggini E, Huth C, Aulchenko YS, Thorleifsson G, McCulloch LJ, Ferreira T, Grallert H, Amin N, Wu G, Willer CJ, Raychaudhuri S, McCarroll SA, Langenberg C, Hofmann OM, Dupuis J, Qi L, Segrè AV, van Hoek M, Navarro P, Ardlie K, Balkau B, Benediktsson R, Bennett AJ, Blagieva R, Boerwinkle E, Bonnycastle LL, Bengtsson Boström K, Bravenboer B, Bumpstead S, Burtt NP, Charpentier G, Chines PS, Cornelis M, Couper DJ, Crawford G, Doney ASF, Elliott KS, Elliott AL, Erdos MR, Fox CS, Franklin CS, Ganser M, Gieger C, Grarup N, Green T, Griffin S, Groves CJ, Guiducci C, Hadjadj S, Hassanali N, Herder C, Isomaa B, Jackson AU, Johnson PRV, Jørgensen T, Kao WHL, Klopp N, Kong A, Kraft P, Kuusisto J, Lauritzen T, Li M, Lieverse A, Lindgren CM, Lyssenko V, Marre M, Meitinger T, Midthjell K, Morken MA, Narisu N, Nilsson P, Owen KR, Payne F, Perry JRB, Petersen A-K, Platou C, Proença C, Prokopenko I, Rathmann W, Rayner NW, Robertson NR, Rocheleau G, Roden M, Sampson MJ, Saxena R, Shields BM, Shrader P, Sigurdsson G, Sparsø T, Strassburger K, Stringham HM, Sun Q, Swift AJ, Thorand B, Tichet J, Tuomi T, van Dam RM, van Haeften TW, van Herpt T, van Vliet-Ostaptchouk JV, Walters GB, Weedon MN, Wijmenga C, Witteman J, Bergman RN, Cauchi S, Collins FS, Gloyn AL, Gyllensten U, Hansen T, Hide WA, Hitman GA, Hofman A, Hunter DJ, Hveem K, Laakso M, Mohlke KL, Morris AD, Palmer CNA, Pramstaller PP, Rudan I, Sijbrands E, Stein LD, Tuomilehto J, Uitterlinden A, Walker M, Wareham NJ, Watanabe RM, Abecasis GR, Boehm BO, Campbell H, Daly MJ, Hattersley AT, Hu FB, Meigs JB, Pankow JS, Pedersen O, Wichmann H-E, Barroso I, Florez JC, Frayling TM, Groop L, Sladek R, Thorsteinsdottir U, Wilson JF, Illig T, Froguel P, van Duijn CM, Stefansson K, Altshuler D, Boehnke M, McCarthy MI, MAGIC investigators, GIANT Consortium (2010). Twelve type 2 diabetes susceptibility loci identified through large-scale association analysis. Nature Genetics.

[bib71] Wilkins JF, Haig D (2003). What good is genomic imprinting: the function of parent-specific gene expression. Nature Reviews. Genetics.

[bib72] Wolf JB, Cheverud JM (2009). A framework for detecting and characterizing genetic background-dependent imprinting effects. Mammalian Genome.

[bib73] Yee TW (2010). The VGAM Package for Categorical Data Analysis. Journal of Statistical Software.

[bib74] Yeh Y-S, Jheng H-F, Iwase M, Kim M, Mohri S, Kwon J, Kawarasaki S, Li Y, Takahashi H, Ara T, Nomura W, Kawada T, Goto T (2018). The Mevalonate Pathway Is Indispensable for Adipocyte Survival. IScience.

[bib75] Young HS, Won HK, Moon C, Yun HH, Eun SY, Joo HL, Joo SC, Song J, Jung MH (2005). Ectopic expression of Neuronatin potentiates adipogenesis through enhanced phosphorylation of cAMP-response element-binding protein in 3T3-L1 cells. Biochemical and Biophysical Research Communications.

[bib76] Zaky DS, Ali AA, Abd-Elraheem SE, Abdel-Moniem SH (2022). Sapub.

[bib77] Zappia L, Oshlack A (2018). Clustering trees: a visualization for evaluating clusterings at multiple resolutions. GigaScience.

[bib78] Zeng Y, Amador C, Xia C, Marioni R, Sproul D, Walker RM, Morris SW, Bretherick A, Canela-Xandri O, Boutin TS, Clark DW, Campbell A, Rawlik K, Hayward C, Nagy R, Tenesa A, Porteous DJ, Wilson JF, Deary IJ, Evans KL, McIntosh AM, Navarro P, Haley CS (2019). Parent of origin genetic effects on methylation in humans are common and influence complex trait variation. Nature Communications.

[bib79] Zernichow L, Åbrink M, Hallgren J, Grujic M, Pejler G, Kolset SO (2006). Serglycin Is the Major Secreted Proteoglycan in Macrophages and Has a Role in the Regulation of Macrophage Tumor Necrosis Factor-α Secretion in Response to Lipopolysaccharide. Journal of Biological Chemistry.

[bib80] Zhang B, Kirov S, Snoddy J (2005). WebGestalt: an integrated system for exploring gene sets in various biological contexts. Nucleic Acids Research.

[bib81] Zheng GXY, Terry JM, Belgrader P, Ryvkin P, Bent ZW, Wilson R, Ziraldo SB, Wheeler TD, McDermott GP, Zhu J, Gregory MT, Shuga J, Montesclaros L, Underwood JG, Masquelier DA, Nishimura SY, Schnall-Levin M, Wyatt PW, Hindson CM, Bharadwaj R, Wong A, Ness KD, Beppu LW, Deeg HJ, McFarland C, Loeb KR, Valente WJ, Ericson NG, Stevens EA, Radich JP, Mikkelsen TS, Hindson BJ, Bielas JH (2017). Massively parallel digital transcriptional profiling of single cells. Nature Communications.

