# Supplementary material for: Parent-of-origin effects propagate through networks to shape metabolic traits
Source: eLife. 2022 Mar 31;11:e72989. doi: 10.7554/eLife.72989 (PMC9075957; doi:10.7554/eLife.72989)
Supplement: Transparent reporting form [file elife-72989-transrepform1.docx]

***eLife’s* transparent reporting form**

We encourage authors to provide detailed information *within their submission* to facilitate the interpretation and replication of experiments. Authors can upload supporting documentation to indicate the use of appropriate reporting guidelines for health-related research (see [EQUATOR Network](http://www.equator-network.org/%20)), life science research (see the [BioSharing Information Resource](https://biosharing.org/)), or the [ARRIVE guidelines](http://www.plosbiology.org/article/info:doi/10.1371/journal.pbio.1000412) for reporting work involving animal research. Where applicable, authors should refer to any relevant reporting standards documents in this form.

If you have any questions, please consult our Journal Policies and/or contact us: [editorial@elifesciences.org](mailto:editorial@elifesciences.org).

**Sample-size estimation**

- You should state whether an appropriate sample size was computed when the study was being designed
- You should state the statistical method of sample size computation and any required assumptions
- If no explicit power analysis was used, you should describe how you decided what sample (replicate) size (number) to use

Please outline where this information can be found within the submission (e.g., sections or figure legends), or explain why this information doesn’t apply to your submission:

RNAseq sample size was chosen to balance financial constraints with the number of diet-by-sex contexts for each reciprocal cross (total of 32 libraries). Due to the complexity of our contexts and various uses of these data, an a priori power analysis would have been unreliable.

**Replicates**

- You should report how often each experiment was performed
- You should include a definition of biological versus technical replication
- The data obtained should be provided and sufficient information should be provided to indicate the number of independent biological and/or technical replicates
- If you encountered any outliers, you should describe how these were handled
- Criteria for exclusion/inclusion of data should be clearly stated
- High-throughput sequence data should be uploaded before submission, with a private link for reviewers provided (these are available from both GEO and ArrayExpress)

Please outline where this information can be found within the submission (e.g., sections or figure legends), or explain why this information doesn’t apply to your submission:

1. Overall experiment was performed once.
2. Biological replicates are samples from different animals. Technical replicates are multiple samples from one animal. Technical replicates were only used for qPCR validation of expression patterns. The value for each biological replicate was taken as the mean of its corresponding technical replicates.
3. When the number of replicates for a given test was fixed, it is reported in the methods section for that test. For tests where sample size was variable due to low gene expression, minimum sample size is indicated for each test ranging from N=4 to N=31.
4. Library complexity was used to identify outliers for ASE detection. A single library had complexity that was too low and was considered an outlier (Supplemental figure 3). When a test required assumptions of normality, values were tested for normality by Shapiro-Wilks test. If data showed a skewed lognormal distribution, these data were log transformed to meet normality assumptions. Any such transformations are reported in methods sections for tests that required them and are demonstrated in Supplemental Figures 12 & 15. Secondary to transformations, samples with values more than 2 standard deviations from the mean (normally distributed data) were considered outliers.
5. All samples that met distributional and library complexity assumptions were included.
6. Sequence data accession given in methods

**Statistical reporting**

- Statistical analysis methods should be described and justified
- Raw data should be presented in figures whenever informative to do so (typically when N per group is less than 10)
- For each experiment, you should identify the statistical tests used, exact values of N, definitions of center, methods of multiple test correction, and dispersion and precision measures (e.g., mean, median, SD, SEM, confidence intervals; and, for the major substantive results, a measure of effect size (e.g., Pearson's r, Cohen's d)
- Report exact p-values wherever possible alongside the summary statistics and 95% confidence intervals. These should be reported for all key questions and not only when the p-value is less than 0.05.

Please outline where this information can be found within the submission (e.g., sections or figure legends), or explain why this information doesn’t apply to your submission:

1. Statistical analysis methods are described in detail in methods section for each statistical test.
2. Raw data are superimposed on boxplots in figures when informative. In the case of figures which do not show individuals, Individual data values are provided in the supplements.
3. Statistical tests are reported in methods sections. Multiple test correction strategies are explained in methods sections in which they are used. Null distributions for permutation based multiple test corrections are plotted in supplemental figures. Thresholds are reported in methods sections and plotted in supplemental figures. Statistics are provided in supplemental tables broken up by test. All test statistics are reported in supplemental tables, including those which were not significant.

(For large datasets, or papers with a very large number of statistical tests, you may upload a single table file with tests, Ns, etc., with reference to sections in the manuscript.)

**Group allocation**

- Indicate how samples were allocated into experimental groups (in the case of clinical studies, please specify allocation to treatment method); if randomization was used, please also state if restricted randomization was applied
- Indicate if masking was used during group allocation, data collection and/or data analysis

Please outline where this information can be found within the submission (e.g., sections or figure legends), or explain why this information doesn’t apply to your submission:

Animals were randomized onto high and low fat diets, described in methods.

**Additional data files (“source data”)**

- We encourage you to upload relevant additional data files, such as numerical data that are represented as a graph in a figure, or as a summary table
- Where provided, these should be in the most useful format, and they can be uploaded as “Source data” files linked to a main figure or table
- Include model definition files including the full list of parameters used
- Include code used for data analysis (e.g., R, MatLab)
- Avoid stating that data files are “available upon request”

Please indicate the figures or tables for which source data files have been provided:

12 Supplemental Tables, information provided in the methods includes:

Raw reads submitted to SRA (accession PRJNA753198)

Code for analysis available:

<https://github.com/LawsonLab-WUSM/POE_Epistasis>
